# Age-Related Neurodegenerative Diseases: A Stem Cell’s Perspective

**DOI:** 10.3390/cells14050347

**Published:** 2025-02-27

**Authors:** Belén Calvo, Pierre Schembri-Wismayer, María Beatriz Durán-Alonso

**Affiliations:** 1 Faculty of Health Sciences, Catholic University of Ávila, 05005 Ávila, Spain; belen.calvo@ucavila.es; 2 Department of Anatomy, Faculty of Medicine and Surgery, University of Malta, MSD 2080 Msida, Malta; pierre.schembri-wismayer@um.edu.mt; 3 Department of Biochemistry and Molecular Biology and Physiology, Faculty of Medicine, University of Valladolid, 47005 Valladolid, Spain

**Keywords:** neurodegenerative disorders, neural stem cells, pluripotent stem cells, neurogenesis, cell models, stem-cell-based therapy

## Abstract

Neurodegenerative diseases encompass a number of very heterogeneous disorders, primarily characterized by neuronal loss and a concomitant decline in neurological function. Examples of this type of clinical condition are Alzheimer’s Disease, Parkinson’s Disease, Huntington’s Disease and Amyotrophic Lateral Sclerosis. Age has been identified as a major risk in the etiology of these disorders, which explains their increased incidence in developed countries. Unfortunately, despite continued and intensive efforts, no cure has yet been found for any of these diseases; reliable markers that allow for an early diagnosis of the disease and the identification of key molecular events leading to disease onset and progression are lacking. Altered adult neurogenesis appears to precede the appearance of severe symptoms. Given the scarcity of human samples and the considerable differences with model species, increasingly complex human stem-cell-based models are being developed. These are shedding light on the molecular alterations that contribute to disease development, facilitating the identification of new clinical targets and providing a screening platform for the testing of candidate drugs. Moreover, the secretome and other promising features of these cell types are being explored, to use them as replacement cells of high plasticity or as co-adjuvant therapy in combinatorial treatments.

## 1. Introduction

Neurodegenerative diseases (NDs) are a highly heterogeneous group of devastating clinical disorders with a very high incidence worldwide; they are primarily characterized by the degeneration and loss of existing neurons, with a concomitant decline in neurological function [1,2]. Although the loss of proteostasis and the accumulation of aberrant protein aggregates are common pathological traits of these diseases [1,3,4,5,6], there is a general lack of reliable biological markers that precludes an early diagnosis. Therefore, most cases are identified long after the first pathological alterations have taken place, thus hindering the identification of the molecular mechanisms leading to disease [7,8]. Patients often present a series of motor symptoms in combination with non-motor symptoms, such as depression, sleep disturbances, anxiety and cognitive impairment. Clear examples of these disorders are Alzheimer’s Disease (AD), Parkinson’s Disease (PD), Huntington’s Disease (HD) and Amyotrophic Lateral Sclerosis (ALS), of which AD and PD are the most prevalent. AD is the leading cause of dementia worldwide with the most affected brain areas being the basal forebrain and the limbic system, and patients present memory and cognitive impairment. At the cellular level, there is a severe impairment in mitochondrial biogenesis and function, extensive gliosis, synaptic dysfunction and neuronal loss; the patients present an accumulation of extracellular plaques of the amyloid beta (Aβ) peptide, cleaved from the β-amyloid precursor protein (APP), and intracellular neurofibrillary tangles (NFTs) consisting of a hyperphosphorylated form of the microtubule-associated protein Tau. These alterations may be preceded by demyelination and the impaired clearance of myelin debris by microglia, which becomes toxic to the neurons [9].

PD patients primarily lose dopaminergic (DA) neurons in the substantia nigra pars compacta and present cognitive and other non-motor impairments, such as anosmia, anxiety and depression, which often precede the onset of severe motor symptoms, including tremor, rigidity, bradykinesia and postural instability [10,11]. A characteristic feature of PD is the formation of cytoplasmic inclusions of aggregated α-synuclein (α-syn) proteins; this is a presynaptic protein that participates in synaptic remodeling and maintenance during embryonic neuronal development, and it is present in regions of adult neurogenesis in the adult brain. Aberrant α-syn aggregates disrupt mitochondrial homeostasis, interfere with normal synaptic transmission and promote neuroinflammation.

HD and ALS patients present progressive motor impairment. The latter suffer from atrophy and muscular paralysis, resulting from the progressive death of upper and lower MNs (MNs) [12], associated with the accumulation of cytoplasmic RNA and/or protein aggregates that result in a loss of function or a toxic gain of function of some proteins [13,14,15,16,17,18,19]. Most patients die 2–5 years after disease onset, generally due to respiratory failure. HD patients also present cognitive and mental alterations that often precede altered motor control. A hallmark of the disease is the presence of intracellular aggregates of a mutated form of the Huntingtin (HTT) protein (mHTT) that contains an aberrant N-terminal polyglutamine stretch of varying length, coded by a polymorphic cytosine–adenine–guanine (CAG) repeat expansion in exon 1 of the HTT gene (also called IT15) [20,21]; the altered sequence causes misfolding and aggregation of the protein [22], ultimately resulting in the selective loss of striatal medium spiny neurons, which is the cell type most vulnerable to the accumulation of the mutant protein [21]. The mutation presents an autosomal dominant mode of action, making HD a genetic disease, in contrast to the majority of cases of ND, where known genetic factors are not considered to play such a determining role [23].

## 2. Evidence of Aberrant In Vivo Neurogenesis in Neurodegenerative Diseases

A whole array of processes have been identified as contributing to the pathology behind the various NDs [4,24,25], including inflammation [26,27], accompanied by astrocyte and microglia activation, disruption of the blood–brain barrier (BBB) [28,29,30], mitochondrial dysfunction [31], excitotoxicity and impaired glucose homeostasis [32,33]. Other processes that have been associated with NDs are epigenetic alterations [16], impaired autophagy that may preclude the removal of abnormal proteins and favor the build-up of protein aggregates [34,35], oxidative stress, aberrant lipid metabolism [36,37,38] and gut dysbiosis [25,28,39]. Nonetheless, the precise roles that some of these factors play in the etiology and progression of NDs have not been clearly elucidated, as exemplified by the limited success achieved in clinical trials aimed at clearing Aβ peptide fibrils, long considered to be the basis of AD pathogenesis [24,27].

Age is a clear risk factor for ND; many of the alterations reported in NDs are observed during aging, such as changes in normal adult neurogenesis [31,40]. Although adult neurogenesis persists throughout life, it decreases during aging [41,42,43,44]. Initially, the subventricular zone (SVZ) that lines the lateral wall of the ventricles and the subgranular zone (SGZ) of the hippocampal dentate gyrus (DG) were considered to be the only neurogenic stem cell niches [1,40], regions where the intercellular interactions and the microenvironmental conditions tightly control the processes of the self-renewal and differentiation of neural stem cells (NSCs) to specific neural cell types. However, neurogenesis (also, gliogenesis) has been subsequently observed in several other locations, such as the spinal cord [28,45], olfactory epithelium, striatum, amygdala and circumventricular organs [11,46,47,48]. Proliferating neuroblasts that are generated in the SVZ express PSA-NCAM (polysialylated neural cell adhesion molecule) and other proteins, such as the microtubule binding protein DCX (doublecortin), and in animal models they have been seen to migrate towards the olfactory bulb (OB) [46]. Neuroblasts formed from stem cells in the SGZ express DCX and other neuronal markers, such as ASCL-1 (achaete-scute homolog-1), and move to the dentate granule cell (DGC) layer; immature progenitors reach their final differentiation stage at their target tissues. An age-related reduction in neurogenesis has been associated with various processes, such as decreased vascularization of the neurogenic niches [49], with reduced access of the NSC populations to blood-born factors that regulate their proliferation and differentiation [50]. The release of some factor that may be deleterious to NSC proliferation into the bloodstream in older individuals has also been discussed [49]. Studies carried out by Wu and colleagues (2023) [51] have shown that neurogenesis diminishes by around 80% in the hippocampus of 8-month-old mice compared to that in 2-month-old controls, and it thereafter continues at a low level. Reduced neurogenesis in aging mice has been associated with changes in the pattern of divisions and the quiescent status of the stem cell pools that lead towards their depletion [52]. Of note, a decrease in the numbers of quiescent stem cells and DCX^+^ progenitors has been observed in human subjects of an advancing age, correlating with their cognitive status [11,44,53,54].

There is supporting evidence that impaired neurogenesis may partly explain the memory, emotional and cognitive deficits, as well as the motor disabilities seen in patients who suffer from NDs [55,56]. Genetic and proteomic analyses have identified whole sets of neurogenesis-related genes and proteins as highly differentially regulated sequences when comparing ND patients to healthy controls [38,57,58].

### 2.1. Altered Neurogenesis in Alzheimer’s Disease

The accumulation of protein aggregates in AD has been linked to suppression of the proliferation of NSCs/progenitor cells and an impairment in the maturation of newly generated neurons, together with an induction of apoptosis. Besides the reduction in numbers of proliferating DCX^+^ cells registered in AD patients, lower cognitive scores have been associated with higher numbers of proliferating NESTIN^+^ SOX2^+^ cells, pointing to a differential effect of disease progression on different populations of stem/progenitor cells [54]. Furthermore, technological advancements in deep learning approaches have allowed for a more profound analysis of the brain proteome and the identification of differences in protein expression programs that are activated in AD patients depending on their age. Thus, younger AD patients have demonstrated a greater increase in the levels of proteins involved in cellular differentiation and early development (e.g., VIM, MAPK1, MAPK3), pointing to the possible de-differentiation of neurons into progenitor cells and the reactivation of a developmental program [59].

Alterations in adult hippocampal neurogenesis have been demonstrated in various animal models of AD, such as the PDAPP mouse model that overexpresses a mutant form of the APP protein and presents the age-dependent accumulation of Aβ plaques [60]; the reduction in SGZ neurogenesis, associated with an abnormal maturation of newly generated neurons, parallels the appearance of behavioral deficits in these animals. Yenkoyan and co-workers (2022) [61] demonstrated increased proliferation indexes in the hippocampus and SVZ of Sprague–Dawley rats following the intracerebroventricular injection of Aβ aggregates; the authors argued that the observed neurogenesis might well be a response to neurodegeneration, even though the generated progenitors did not completely mature into neuronal and glial cell types and ultimately resulted in higher rates of neural cell loss; functional tests evidenced cognitive decline and increased anxiety levels in these animals. Impaired neurogenesis has also been reported in the APP/PS1 (presenilin-1) mouse model of AD [62,63]. MicroRNAs (miRNAs) have been shown to play a key role in regulating adult hippocampal neurogenesis, and the miRNA-mediated regulation of NSC behavior and neurogenesis by APP has been described [64]; increased levels of miR-146a-5p have been identified in the APP/PS1 model, and the administration of an antagonist to this miRNA led to the rescue of neurogenesis in these animals [63].

In another series of studies, Hollands and colleagues (2017) [55] reported exacerbated learning and memory deficits in APP/PS1 mice following ablation of adult neurogenesis. Higher levels of phosphorylated Tau protein were recorded in DCX+ neuroblasts and in newly forming neurons in the hippocampus of these mice, likely hindering neuronal maturation and leading to a clear decrease in immature DCX+ neurons; no changes were registered in the levels of full-length APP or oligomeric Aβ protein [55].

It has been shown that alterations in the size of the stem cell and progenitor cell pools, alongside a reduction in adult neurogenesis, may already take place at the pre-symptomatic stages of ND [44,54,65,66]. In this regard, Moreno-Jiménez and co-workers (2019) [44] have reported progressively reduced numbers of DCX^+^ cells and an impairment in the maturation of these cells in AD patients who are at the earliest Braak stages, even before amyloid plaques and NFTs are present. Impaired neurogenesis that precedes the formation of amyloid plaques and other neurodegenerative hallmarks of AD has also been recorded in the SVZ and OB areas. Studies on Tg2576 mice, a model of AD that overexpresses a mutated form of human APP, indicated that there is a reduction in the numbers of proliferating NSCs in the SVZ of these mice at the presymptomatic stages of AD [67]. On the other hand, increased numbers of DCX^+^ neuroblasts were reported in these mutant mice, as opposed to a decrease in the size of the SOX2^+^ GFAP^+^ population, resulting from a bias of the NSCs in the SVZ to differentiate towards the neuronal lineage, together with a halt in neuroblast differentiation. The outcome was the generation of fewer and poorly differentiated neurons and of astrocytes that exhibited an altered morphology that resembled that of neurotoxic reactive A1 astrocytes. Neurogenesis was also impaired in the OB, where the population of newborn NeuN^+^BrdU^+^ neurons was diminished, and poorly differentiated neurons were observed. Impaired neurogenesis correlated with an accumulation of intracellular Aβ oligomers in adult NSCs, since treatment with a conformation-specific antibody to these oligomers resulted in normal NSC proliferation rates and yielded differentiation patterns resembling those of controls [67].

Reduced NSC/NPC proliferation and neurogenic activity were also recorded in neurogenic niches in triple-transgenic Alzheimer’s Disease (3xTg-AD) mice, already at postnatal stages in the SGZ and prior to the appearance of amyloid plaques and NFTs [36,66]; in addition, the abnormal accumulation of lipid droplets in the SVZ niche was observed [68]. Alterations in lipid metabolism have been reported in AD and other ND patients [37,38,68].

### 2.2. Altered Neurogenesis in Parkinson’s Disease

Suppressed stem/progenitor cell proliferation has been recorded in the SVZ of PD patients and animal models, accompanied by altered cell differentiation patterns. On the other hand, increased numbers of proliferative neuroblasts and DCX+ immature DGCs that exhibit impaired maturation have been observed. Deficits in stem cell proliferation and differentiation appear to correlate with reduced dopaminergic input during disease progression. Lower progenitor survival rates have also been described.

Data obtained from animal models of PD agree with the reports on significantly decreased numbers of proliferating cells and immature neurons in the DG and other neurogenic brain regions of PD patients. Work conducted by Crews and collaborators (2008) [69] established an association between the decreased survival of neural progenitor cells (NPCs) in the SGZ of the hippocampus of transgenic mice that overexpressed a mutant form of α-syn and reduced Notch signaling. A transgenic rat model of PD that carries a human full-length α-syn sequence, generated by Kohl and co-workers (2016) [65], exhibits severely reduced dendritogenesis and alterations in the expression patterns of various synaptic proteins and markedly lower survival rates of hippocampal DCX^+^ neuroblasts, preceding the loss of DA neurons and the onset of motor deficits; a possible link has been suggested between these alterations and deficits in the serotonergic input to the hippocampus [65] that have been associated with the early non-motor symptoms of PD, such as anxiety and depression. Studies on another transgenic mouse model of PD by Jiang and colleagues (2021) [70] have established a link between hyperphosphorylation of the amyloid precursor protein APP and impaired hippocampal neurogenesis, characterized by reduced NSC numbers, neural proliferation and differentiation; the model expresses a mutated form of the vacuolar protein sorting protein 35 (VPS35) that is associated with late-onset autosomal dominant PD. Receptors for the monoamine neurotransmitter dopamine have been identified in neurogenic niches, and adult neurogenesis in the hippocampus, SVZ and substantia nigra is likely partly regulated by dopamine; larger numbers of surviving DA neurons have been associated with improved motor responses in rodent models of PD. Furthermore, stimulation of D1-dopamine receptors in the hippocampus of Parkinsonian rats leads to the activation of Wnt/β-catenin signaling, which plays a key role in the regulation of NSC self-renewal and differentiation and ameliorates non-motor symptoms, such as anxiety and depression-like phenotypes [71].

### 2.3. Altered Neurogenesis in Huntington’s Disease

Reduced neurogenesis has been recorded in the hippocampus of HD patients that may be responsible for their early spatial learning deficits. However, contrary to what has been described in PD, a thicker SVZ has been observed in postmortem HD brains, as a result of increased cell proliferation, which has been considered a possible neuroprotective response [72,73,74]; the proliferating cells have been identified as neuronal and glial cell progenitors, according to their expression of βIII-Tubulin and GFAP proteins, respectively [46,75]. Impaired maturation and altered migration and differentiation patterns of neuroblasts have also been described.

Reduced rates of NSC proliferation have been observed in the hippocampus of most genetic animal models of HD [72]. Simpson and colleagues (2011) [76] reported decreased neurogenesis and impaired neuronal differentiation in the DG of the transgenic YAC128 mouse model of HD; these alterations were not observed in the SVZ. Additionally, work carried out by Kandasamy and co-workers (2015) [77] on a transgenic rat model of HD did not identify any defect in the hippocampus DG of 8-month-old animals while reduced cell proliferation rates were observed in 12-month-old rats, pointing at age and/or pathological states as factors contributing to disease progression in this model [73,77]. Reduced neurogenesis was also demonstrated in the hippocampus of C9orf72-knock-out mice that exhibited diminished expression levels of the longevity gene Klotho in their DG [78,79].

Recordings of altered neurogenesis may also correspond to changes in the migration patterns of neural precursors. Kandasamy and co-workers (2015) [77] described a reduction in cell proliferation in the SVZ and OB of a transgenic rat model of late-onset HD (tgHD) that runs parallel to enlarged numbers of DCX^+^ progenitors; this was explained by many proliferating DCX^+^ cells migrating to the damaged striatum. The increased cell proliferation and migration of SVZ progenitors towards non-neurogenic damaged areas has been observed in other rat models of HD and in other pathological conditions, such as ALS; it has been regarded as a failed attempt at regeneration, since the migrating cells do not reach full maturity or restore the function of the degenerating neuronal circuits [11,75,80,81]. In addition to changes in the proliferation of stem and progenitor cell compartments and in the migratory paths the newly generated cells follow, alterations have also been recorded in NPC differentiation patterns [82,83]; as an example, differently from what is observed in control animals, NPCs present in the OB of tgHD rats primarily differentiate towards the dopaminergic lineage, likely to be less susceptible to the accumulation of mutant HTT protein [77].

### 2.4. Altered Neurogenesis in Amyotrophic Lateral Sclerosis

Studies on adult hippocampal neurogenesis have shown that there is an increase in the numbers of NSCs and DCX^+^ immature DGCs in ALS patients [40]. Increased cell densities are coupled to an impaired capacity for development that results in higher apoptotic rates.

In a series of studies, Verdile and colleagues (2023) [84] recorded an early amplification of NPC proliferation rates in an hSOD1^G93A^ transgenic mouse model of ALS, which was accompanied by an impairment in their differentiation capacity. Additionally, the responses of different neurogenic niches to a neurodegenerative process may differ [76]. Galán et al. (2017) [85] have described a significant increase in the proliferation of SVZ cells in ALS patients, coupled to a reduction in the proliferation of SGZ GFAPδ neural pluripotent cells. These changes in neurogenesis correlate with the levels of cytoplasmic pTDP-43 (phosphorylated transactivator regulatory DNA-binding protein 43), one of the hallmarks of ALS, considered by some as a marker of disease progression [85].

## 3. Stem Cell-Based Models of Neurodegenerative Diseases

There are a series of important hurdles to the study of neurodegeneration in human patients. Firstly, there is a shortage of tissue samples from these patients; moreover, for them to be a reliable source of information, extreme care must be applied during tissue collection and processing [44]. Tissue is mostly obtained following the demise of the individual, precluding the identification of changes that may have occurred during disease progression. The elucidation of the mechanisms involved in disease development is thus greatly benefiting from the use of animal models [67]. Nevertheless, there are important constraints to the use of such models, such as inter-species differences in the expression and cellular regulation of key proteins and the high degree of genetic and phenotypic heterogeneity that characterize NDs [86,87,88]. Very frequently, the studies are carried out on rodent models [86], taking advantage of their greater amenability to undergo genetic modification and the possibility to introduce mutations in their genome that are characteristic of familial cases of ND. It is sometimes necessary to introduce the mutated human gene, since mutations in the animal’s orthologue do not yield the expected outcome [89]. Nonetheless, NDs are highly complex processes that likely result from the interaction of genetic and environmental factors and are mostly sporadic [90]; thus, genetically generated animal models mirror some of the symptoms in the patients but may miss what could be key clinical targets. Furthermore, the lifespan of animals, such as rodents, clearly differs from that of humans, and they do not adequately replicate aging and the various stages of disease in the patients. Other models, such as non-human primates, exhibit phenotypes that more closely mimic the symptoms seen in humans but are too costly. NDs have an enormously negative impact on patients’ lives; given their high incidence, it is imperative to develop new models that help gain a deeper insight into these pathologies so that early diagnostic markers and new therapeutic targets may be identified.

Desirable *in vitro* models should closely replicate the high complexity encompassed by human ND while allowing for the manipulation of the genetic and environmental conditions, the identification of individual players in the pathology and the elucidation of their roles at the various stages of disease [16,91,92,93]. Importantly, NDs are often the outcome of alterations in more than one cell type and/or the interaction among various cell types [94,95,96,97]. For example, although the hallmark of ALS is the loss of MNs, it has been shown that glial cells secrete factors that are toxic to those neurons, and it is therefore necessary to generate cellular models where both cell types co-exist and interact [94,98]. This is also applicable to microglia, key elements in the regulation of synaptic pruning, neuronal maturation and inflammatory responses. Furthermore, the presence of other non-neural cell types, such as vascular endothelial cells and choroid plexus epithelial cells, is also to be pursued, since these constitute barriers to the nervous tissue and regulate many processes, such as the crossing to the brain of neurotrophic and neuroprotective factors, inflammatory responses and the biogenesis of extracellular vesicles for secretion [99,100,101,102]; the effects of the vascular endothelium and the choroid plexus on the behavior and proliferation status of neural progenitors have been described [50,103,104].

### 3.1. NPCs

Highly interesting cellular models are those based on adult NPCs that are isolated from the post-mortem tissue [105] of patients with NDs, as shown by Wang and colleagues (2012) [106]; these researchers identified alterations in the ability of PD adult NPCs to differentiate into neurons and glia under standard *in vitro* conditions. Nonetheless, the starting material to create these models is very scarce, and long-term cultures are difficult to maintain [106]. There are however some commercially available, immortalized human NPC (hNPC) lines, derived from embryonic tissue [100,107,108].

### 3.2. iPSCs

One of the best alternatives to generate ND models consists in the use of human induced pluripotent stem cells (hiPSCs). Their amenability to differentiate into different cell types is comparable to that of embryonic stem cells (ESCs) and exceeds the differentiation abilities of other stem cells, such as the mesenchymal stem cells (MSCs). In comparison to NPCs, with a more restricted differentiation potential, iPSCs can give rise to multiple cell lineages, of a neural and non-neural origin.

iPSCs may carry specific ND-related mutations that are introduced into their genome using genome-editing tools, such as the CRISPR/Cas9 system (clustered regularly interspaced short palindromic repeats/CRISPR-associated system 9) [109]; alternatively, they may be directly generated from the patients themselves, irrespective of their age and of whether their disease is linked to a specific genetic mutation or not (i.e., familial (f) *vs.* sporadic (s) condition) [110,111,112]. The possibility of establishing hiPSC lines from somatic cells isolated from elderly patients [113,114] is highly relevant, as most NDs are age-associated. The results obtained with patient-derived hiPSC-based models can be directly compared to any gene expression and physiological data that are available from the donor, so that the reliability of the *in vitro* model may be tested [115] and the observations made on the cultures may be translated to the patient [2]. Contrary to the available animal models, hiPSC-based models carry all the genetic information from the patient, regardless of whether their disease is sporadic or caused by a specific gene mutation. Thus, pathophysiological mechanisms may be unveiled other than those already associated with priorly characterized mutations, providing new therapeutic targets.

hiPSC lines have been differentiated into relatively highly pure cultures containing a single cell type [116] or, alternatively, they have been used to generate mixed cultures, allowing for the analysis of cell–cell interactions that may be affected during the disease [96,117].

Drawbacks to the use of hiPSCs are the long-term culturing required to differentiate these cells, which mostly yield immature cell phenotypes, the variability among clones to yield various cell lineages and the fact that reprogramming donor cells to hiPSC conveys a loss of age-related traits. With regards to the latter point, recent data support the suitability of using hiPSC-derived cell types to study the molecular mechanisms underlying age-related neurodegeneration [2,83,118,119]; in fact, it has been argued that these *in vitro* models lack the mechanisms that delay the onset of neurodegeneration of the human brain [119]. Nonetheless, the aging of hiPSC-derived models has been attained through various methods (for an excellent discussion on these approaches, see [120]), such as the ectopic expression of progerin [121], blocking neddylation [122], low density seeding of the cultures [83] and the application of a combination of factors that affect chromatin remodeling and calcium-dependent transcription (*GENtoniK*), promoting the maturation of hiPSC-derived cell types [123]. Importantly, aging hallmarks in hiPSCs have been shown to synergize with ND-associated gene mutations, giving rise to late-onset disease phenotypes that could not be replicated in the corresponding non-aged models carrying the same mutations [122]. An alternative to the manipulation of hiPSCs to promote the aging of the cultures is the direct reprogramming of aged somatic cells into neurons (iNs), by expressing a series of transcription factors that induce specific neuronal subtypes [124]. Some comparative studies have shown that iNs may better recapitulate the neuropathology of late-onset NDs than hiPSC-derived neurons; however, reprogramming efficiencies when generating iNs are low, and the resulting cells are not expandable, since they are post-mitotic cells; these are severely limiting factors.

The large phenotypic variability exhibited by hiPSC clones makes it difficult to establish a causal relation between some mutations and the neuropathologies under study. Genome edition using CRISPR/Cas9 technology renders patient-derived hiPSC lines that have the same genetic background and only differ in the presence or absence of a mutation so that the causative role of the mutation may be demonstrated [92,98,125,126]; CRISPR-based functional genomic screening has also been carried out to identify disease-modifier genes [127].

Another aspect that is to be considered when establishing hiPSC-derived cell models of neurodegeneration is the long times needed to differentiate these cells towards the lineages of interest so that the aberrant phenotype may be replicated [16,128]. Shorter differentiation times have been achieved by selecting defined reagents to culture hiPSCs [129] and also by introducing transcription factors into the hiPSCs that are specific to the cellular subtype of interest [2,116]. Important shortcomings to this latter approach are that no intermediate cell types are obtained that can shed light on mechanisms participating in disease progression and that the procedure to obtain the differentiated cell types must be re-initiated every time from the hiPSC stage. Protocols have been optimized to establish a large preservable NPC population that may be subsequently differentiated into varying cell types through the introduction of cell-type-specific transcription factors [130,131]. Varying pools of NPCs may be generated, with different regional identities that depend on the inducing factors used to differentiate the hiPSCs towards NPCs; this method can be applied to hiPSCs from patients with different NDs, yielding similar NPC-induction rates [130].

#### 3.2.1. iPSC-Based Organoids

Increasingly, complex cell models have been achieved with hiPSCs in the form of organoids [99,132,133,134,135,136,137,138,139]. These are three-dimensional cultures where a variety of cell types emerge over time and establish cell–cell and cell–matrix interactions that more closely mimic the *in vivo* situation [91]. These favorable features come at a cost, since the growth of the organoid and the maturation of the existing cell types are compromised by the lack of oxygen and nutrients that reach the innermost area of the organoid; this results in apoptosis in the central regions within the organoid and in alterations in the potential of NPCs to proliferate and differentiate into more mature cell types. The problem is being addressed by generating vascularized organoids; experiments carried out by Cakir and co-workers (2019) [99] demonstrated that the development of a vasculature in human cortical organoids supports the growth of the organoid and accelerates the functional maturation of the neurons while preventing cell death in the inner regions. Besides a requirement for technologically more demanding analyses [133], organoids present a higher variability than 2D models; it is now known that NSCs induced during the generation of brain organoids differ in their regional identity; that is, the final cellular composition of the organoid will depend on the method applied to induce NPCs within the forming organoid [140]. A careful selection of the derivation method may yield a model that more adequately replicates the cell types and the pathophysiological processes involved in the disease under study. Further levels of optimization of the ND model come from the creation of assembloids, combinations of organoids that resemble different brain regions, so that the differentiating cells may establish interactions with other cells across the culture that recapitulate the communication across different brain regions that occurs *in vivo* [91]. Undoubtedly, organoids offer one of the closest human models to ND, in terms of the cell composition, cell–cell and cell–matrix interactions in a three-dimensional arrangement and the differentiation to more mature cell phenotypes. Furthermore, cell features may be preserved in organoids that are otherwise very difficult to maintain *in vitro*; this is the case with microglia, which undergo genetic and morphological changes when cultured in standard monolayers [88,141,142].

#### 3.2.2. Use of hiPSCs to Generate Human-Mouse Transplantation Chimaeras

Another application of hiPSCs to create ND models consists in the transplantation of hiPSC-derived cell lineages into murine hosts to generate human–mouse transplantation chimaeras [143,144,145,146]. In this way, the response of specific human cell types can be analyzed in an *in vivo* setting that more closely resembles the normal physiological conditions; it also overcomes some of the problems posed by *in vitro* culture artefacts and by species-specific differences in gene expression and cellular responses *in vivo* [143,147,148]. These models may be used to establish correlations between the role a certain gene isoform or gene mutation plays on disease progression and the cell type that harbors such sequence [144].

A series of stem cell-based models that have been developed for the study of NDs are discussed below; these have been summarized in Table 1.

### 3.3. Alzheimer’s Disease Models

Although some discrepancies have been observed among the different studies, results obtained on stem/progenitor-cell-based models validate the observations made on animal models and post-mortem tissue from AD patients [95,118,138,146,152,154]. Furthermore, *in vitro* models have been used to shed light on processes that could not be unambiguously elucidated in complex *in vivo* settings, such as the relation between Aβ oligomer formation and aberrant Tau processing and the interaction of aging with the genetic components of the disease. Thus, it has been shown that Aβ accumulation is an upstream player in the tauopathy observed in AD [95,107,149] and a likely contributor to other alterations in these patients, such as the increased permeability of the BBB [100]. It has also been demonstrated that age-related processes, such as neddylation inhibition, synergize with AD-related gene mutations to evoke late-onset AD phenotypes, like Tau pathology [122]; these studies provide an explanation for the late onset that characterizes many of these NDs, despite the presence of inborn mutations. Stem-cell-derived AD models are also being used to conduct drug testing studies [100,116,150,159,218] in search for compounds that may revert the aberrant phenotype.

#### 3.3.1. Evidence of Reduced Cell Proliferation and Premature Neuronal Differentiation

Besides the accumulation of Aβ aggregates and NFTs, well-described in a whole range of AD models [118,138,146], reduced proliferation and premature neuronal differentiation have been reported in AD-related stem cell-based models [119,152]. Meyer and colleagues (2019) [152] demonstrated decreased NPC renewal and accelerated neuronal maturation and synapse formation, together with increased electrical excitability in cultures of NPCs derived from hiPSC lines obtained from sporadic AD (sAD) patients. These alterations preceded any amyloid and Tau pathology and were correlated with epigenetic transcriptional dysregulation caused by a reduced translocation of the transcriptional repressor REST (Repressor Element 1-Silencing Transcription Factor) to the nucleus. The accelerated maturation of neurons derived from hiPSC cultures carrying PS1 mutations was also observed by Ghatak and colleagues (2019) [119], while neurons carrying mutations in the APP gene did not exhibit this phenotype. Additionally, studies on a series of 18 hiPSC lines obtained from sAD and fAD patients and from healthy controls evidenced alterations in the differentiation of the AD-derived lines when senescence was induced; in this case, AD-hiPSCs showed an impaired potential to differentiate into neurons and oligodendrocytes and increased differentiation to astrocytes; this was associated with BMP4 hypersecretion and activation of the SMAD1/5/9-RUNX2 signaling pathway [83].

#### 3.3.2. Evidence of Impaired Neuronal Homeostasis

Impaired neuronal homeostasis has been described in stem cell-based AD models. Transcriptome analyses of sAD-hiPSC-derived neuronal cultures have revealed the downregulation of genes related to the ubiquitin–proteasome system [114]; deficiencies in these processes have been described in AD patients and are thought to occur at early stages of the disease, contributing to the build-up of protein aggregates in the cell. A screen for genetic modifiers of Tauopathy by Parra Bravo and co-workers (2024) [127] has identified alterations in genes related to vesicular trafficking, the activity of the retromer complex, autophagy and UFMylation.

#### 3.3.3. Evidence of Alterations in Synaptic Activity

Some studies point to synapse loss as the earliest response to soluble Aβ42 species exposure [95], and alterations in synaptic activity have been reported in neuronal cultures and cerebral organoids generated from hiPSCs carrying AD-related mutations [146]. Ghatak and co-workers (2019) [119] observed decreased neurite lengths and the hyperactivity of neuronal cultures derived from AD-hiPSCs; increased levels of VGLUT1 were recorded in these cultures, supporting an increase in excitatory neurotransmission [119]. In addition, a decrease in inhibitory activity was observed, correlating with a reduction in the numbers of GABAergic neurons [119]. In another series of studies, Targa Dias Anastacio and co-workers (2024) [93] registered increased Ca^2+^ responses to glutamate and AMPA in neuronal cultures derived from hiPSCs carrying PS1 mutations that are present in fAD patients. This calcium dysregulation occurred even in the absence of amyloid and Tau phenotypes.

#### 3.3.4. Studies on the Contribution of Various Cell Types to AD Pathology

Using hiPSC-derived astrocytes and neurons carrying mutations in the *APP* or *PS1* genes, Salcedo and colleagues (2024) [161] registered increased glucose metabolism, associated with higher levels of glutamate being synthesized by these mutated cell types and downregulation of the excitatory amino acid transporter 2 (EAAT2) in the astrocytes, with a concomitant decrease in their capacity for glutamate re-uptake and increased glutamine uptake by the neurons. Other studies have demonstrated that astrocytes near Aβ plaques exhibit a reactive phenotype, with an impairment for glutamate uptake, changes in morphology and the release of pro-inflammatory factors [219,220]; a potential role for astrocytes in the compaction of Aβ has been put forward by Bassil and co-workers (2021) [95].

Many other studies have explored the interactions and contribution of the various cell types to AD pathology [95,96,143,144,146,153]. A role for infiltrating T cells in AD pathology has been demonstrated, with a synergistic effect of CD8^+^ T cells and microglia to promote damage to neurons and astrocytes carrying AD-associated mutations [155]. The recruitment of microglia to 3D co-cultures of neurons and astrocytes differentiated from hNPCs and hiPSC-derived-NPCs that overexpressed pathogenic Aβ species led to an accelerated AD-related pathology and increased rates of neuron and astrocyte loss. Modeling an inflammatory microenvironment in tri-cultures containing neurons derived from control hiPSCs or from hiPSCs expressing a mutated APP isoform, astrocytes and microglia demonstrated that the presence of microglia led to increased levels of inflammatory cytokines, such as IL-6 and TNF-a, and increased production of complement C3; an astrocyte–microglia crosstalk initiated by C3 production by microglia was identified as contributing to augmented C3 secretion [96]. The presence of neurons carrying the mutated APP gene further promoted this increase. Interestingly, Bassil et al. (2021) [95] reported on the ability of hiPSC-derived microglia to internalize soluble Aβ42 species and exocytose compacted forms, initiating plaque formation. Although such activity appeared to exert a neuroprotective effect in neuron–astrocyte–microglia tri-cultures, this effect was lost when the cultures were exposed to a pro-inflammatory environment; overactivation of the microglial response ensued, with cytokine secretion and an increase in plaque formation that became neurotoxic [95]. A contribution of microglia to the formation of Aβ and p-Tau aggregates was described by Rao and co-workers (2025) [163], who registered an effect of neuronal APOE4 on the microglial status. Activation of a population of hiPSC-derived microglia exposed to p-Tau was shown to facilitate the aggregation and propagation of p-Tau, promoting the formation of extracellular vesicles that could transport the altered protein [162].

Increased permeability of the BBB has also been recorded in AD-model cultures of the neurovascular unit that contain stem-cell-derived neurons and astrocytes; these alterations were associated with the dysregulation of key endothelial markers and the presence of Aβ plaques both in the neuronal and in the vascular compartments [30,100,108].

#### 3.3.5. Analysis of AD-Associated Genetic Mutations and Genetic Risk Factors

Stem-cell-based models have been employed to analyze the specific effects of AD-associated genetic mutations and also the genetic risk factors, such as SORL1 (sortilin-related receptor 1), TREM2 (triggering receptor expressed on myeloid cells 2) and APOE4 (apolipoprotein E4) [119,144,148,151,153,159]. APOE4 is the strongest genetic risk factor for sAD; data obtained from cerebral organoids generated from AD-hiPSCs carrying either the APOE3 or the APOE4 genotype indicate that, besides increased levels of Aβ species and p-Tau accumulation, the latter promotes an enhancement of stress granules, disrupted RNA metabolism, apoptosis and decreased synaptic integrity [154]; APOE4-containing AD-hiPSC-derived cultures also exhibit decreased NSC plasticity [221] and accelerated neuronal maturation [154]. Najm and co-workers (2020) [144] showed that, when expressed in hiPSC-derived excitatory neurons, the APOE4 genotype was associated with changes in the transcriptome, synaptic dysfunction and the dysregulation of calcium signaling; interestingly; its effect on inhibitory neurons was different, with a dominant effect on transcriptional responses to UPR (unfolded protein response), oxidative stress and RNA degradation. Thus, a role for the neuronal subtype on APOE4-mediated effects was demonstrated. Importantly, a contribution of the APOE4 signature to neurodegeneration was identified for both endogenous and exogenous APOE4 in a chimeric human–mouse system [144]; differences were observed, depending on whether APOE4 was produced by transplanted hiPSC-derived neurons and/or by the host murine brain [144]. Of note, APOE4-expressing hiPSC-derived neurons only generated Aβ aggregates when transplanted into the murine brain, but not when cultured *in vitro* [144].

When present in hiPSC-derived astrocytes, the APOE4 genotype induces atrophy and increased oxidative stress in these cells, which show altered inflammatory responses and Aβ secretion, an impaired ability to support neuronal survival and a deleterious effect on the neuronal synaptic architecture [96,157]. hiPSC-derived microglia carrying the APOE4 isoform or mutations in the TREM2 gene demonstrate an impaired ability to respond to Aβ plaque formation [144,148,153,160]. Interestingly, it has been shown that there are at least two distinct phases in the microglial response to Aβ accumulation (Human Leukocyte Antigen state and cytokine/chemokine cytokine response), which appear to play opposing roles during disease progression; these states are differentially modulated by APOE4 and by TREM2 [148]. TREM2 mutations have been associated with a proinflammatory gene expression signature of microglia and exacerbated pruning activity of these cells [160]. The altered migration and phagocytic and metabolic activity of hiPSC-derived microglia carrying the APOE4 genotype have been described, together with an increase in cytokine secretion [66,151]. APOE4 also exerts an effect on the BBB; studies by Ding and co-workers (2024) [158] have shown that, while the APOE2 genotype appears to confer some degree of protection against Aβ accumulation, an APOE4 genotype in hiPSC-derived brain microvascular endothelial cell-like cells (BMECs) and in hiPSC-derived pericyte-like cells is associated with decreased Aβ42 clearance by the former cell type and increased extracellular Aβ42 deposition by the latter, all contributing to a higher amyloid load [158].

### 3.4. Parkinson’s Disease Models

PD-hiPSC lines have been generated from patients’ cells and used to validate the observations made on animal models and patients’ tissue, as well as to investigate correlations between different elements that would otherwise be difficult to study in the living organism [222,223]. An example of this is the analysis of Lewy body inclusions.

#### 3.4.1. Studies on α-Synuclein Aggregation

Bayati et al. (2024) [168] studied the formation and composition of Lewy body inclusions by sequentially exposing hiPSC-derived DA neurons to α-syn fibrils and then to an immune challenge. These inclusions were also formed when α-syn-overexpressing neurons were treated with IFN-γ. The effect was specific to DA cells since it was not observed with hiPSC-derived cortical neurons or other cell types; it was accompanied by the downregulation of lysosomal proteins, such as TFEB, NRF2, LAMP1 and LAMP2, lysosomal impairment and dysfunctional autophagy, which contributed to aggregate build-up. Similarly, the co-culture of hiPSC-derived DA neurons with an activated human microglia-like cell line promoted inclusion formation in the neurons, contrary to the effect exerted by their co-culture with astrocytes, which appeared to reduce the neuroinflammatory process and fibril load [168]. Other studies have been conducted on α-syn aggregation into Lewy body inclusions and the propagation of α-syn fibrillary aggregates [171,173]; these processes were found to be promoted by loss-of-function mutations in the GBA1 gene, the most frequent risk factor for PD, encoding the lysosomal glucosylceramide-catalyzing enzyme glucocerebroside [171]. hiPSC-derived DA neurons harboring heterozygote mutations in GBA1 exhibited mammalian target of rapamycin complex 1 (mTORC1) hyperactivity and a block in autophagy [166]. The most common genetic cause of fPD, a mutation in the LRRK2 (Leucine-rich repeat kinase 2) gene (G2019S variant) also promoted α-syn aggregation in a PD-hiPSC-derived neuron model treated with preformed fibrils [164]; the same mutation was associated with defects in the axonal trafficking of lysosomes [170]. Morrone Parfitt and colleagues (2024) [174] reported impaired lysosomal proteolysis in organoids generated from hiPSCs deficient in the protein deglycase DJ1, which is causally associated with early-onset PD; this resulted in the accumulation of advanced glycation end products (AGEs) and α-syn aggregation due to an altered capacity of the astrocytes to clear toxic damaged proteins and to their acquisition of a pro-inflammatory phenotype [174].

#### 3.4.2. Evidence of Alterations in Differentiation, Maturation and Cell Survival

Alterations in the differentiation potential of PD-NPCs have been described. Wang and colleagues (2012) [106] observed that, while PD patient-derived adult NPCs transplanted into murine brains could differentiate into neurons, they could not differentiate into astrocytes under standard *in vitro* conditions. On the other hand, Bernal-Conde and co-workers (2024) [169] reported that, although NPCs derived from PD-hiPSCs with altered SNCA gene expression (over-expression or knock-out expression) could give rise to TH^+^ neurons, the transplantation of these progenitor cells into a pre-clinical PD rat model resulted in decreased numbers of TH^+^ neurons in the transplants, compared to control NPCs. A supportive role of the host tissue in neuronal survival was shown, pointing to a negative impact of altered α-syn levels on DA neuron maturation.

A possible involvement of the engrailed-1 (EN1) gene in PD has been put forward by Hembach and collaborators (2024) [172], since this gene is essential for the development and survival of midbrain DA neurons and is associated with mitochondrial respiration, closely linked to the etiology of PD. Although it did not influence their differentiation into DA neurons, the absence of EN-1 expression in EN-1 KO hiPSC-derived NPCs led to the formation of TH^+^ neurons with shorter neurites that exhibited reduced mitochondrial respiration [172]. Transcriptome analyses of these cultures indicated alterations in processes related to cilia-related processes. Increased WNT signaling was registered, in agreement with the role of primary cilia in regulating signaling transduction pathways, such as those of WNT and SHH; alterations in cilia functionality may thus contribute to the vulnerability of DA neurons in PD [172]. Other PD-associated genes, such as LRRK2 have been linked to the regulation of ciliary function [224].

### 3.5. Huntington’s Disease Models

#### 3.5.1. Evidence of Changes in Gene Expression and Impaired Development and Maturation

Gene expression heterogeneity has been described in HD-hiPSC-derived NPCs, pointing to early changes that may precede ND pathology [191]. Using PSCs from the Rhesus macaque, Goodnight and co-workers (2019) [188] identified changes in chromatin accessibility and transcription during differentiation from PSCs to NPCs and from NPCs to astrocytes; the authors also reported premature activation of the astrocyte differentiation program, as well as altered P53 signaling and E2F dysregulation leading to aberrant cell cycle re-entry and apoptosis in astrocytes. Altered transcriptomics were also reported in HD-hiPSC-derived NSCs [177,225,226], with the differential expression of pathways, such as TGF-β and Netrin-1 [177]. Increased MMP activity and decreased TIMP-1 and TIMP-2 expression were registered in HD-NSCs, pointing at an augmented cleavage of mHTT, increased neurotoxicity and an early disruption of neuronal cell development in HD [226]. Also, hNSCs derived from hESCs carrying HD-associated mutations displayed changes in spindle orientation and the displacement of dynein, dynactin and NuMA (nuclear mitotic apparatus) proteins [178].

Omics analyses of neural cultures derived from HD-hiPSCs and from control hiPSCs have unveiled reduced expression in HD-derived cultures of genes related to glutamate and GABA signaling, axonal guidance and calcium influx [182]. A large proportion of the genes with altered expression are linked to neuronal development and maturation, pointing at an impairment in neurodevelopmental pathways that could render these cells more vulnerable to pathology. DNA methylation changes were recorded in HD-hiPSCs, compared to hiPSCs obtained from healthy controls, with increased methylation during their differentiation to striatal neurons [180]; pathways that were mostly affected were associated with neuronal development and differentiation (e.g., axon guidance, WNT and TGF-β pathways, SMAD and GABA receptor signaling, etc.); additionally, clear hypermethylation at the promoter sequence of the WDR5 (WD repeat-containing protein 5) gene that codes for a chromatin remodeling protein was identified in HD-hiPSCs [180]. Other work on HD-hiPSC-derived cultures has shown that CAG expansion in the HTT gene interferes with the downregulation of OCT4 and with the proper expression of PAX6, followed by a failure of the cells to acquire proper ventral telencephalic progenitor identity and to develop into terminally differentiated striatal neurons [184]; alterations in cortical development and tissue organization were also described in 3D organoids grown from these hiPSC lines, with reduced expression of genes involved in neuronal migration and differentiation [184].

#### 3.5.2. Evidence of Impaired Neuronal Homeostasis

Other features that have been described in HD-hiPSC-derived medium spiny neurons have been increased store-operated channel activity and calcium entry and increased lysosomal numbers and activity [175,179,227,228], defects in synapse structural organization and a large number of spines on dendrites [228], decreased ATP levels and glycolytic activity [176,189] and increased apoptotic rates following BDNF withdrawal [176]. Differences in phenotypes have been identified in HD-hiPSC and HD-hESC lines that are dependent on the length of CAG repeat expansions [176,186]; chromosomal instability and failed cytokinesis leads to impaired neurogenesis, with the emergence of multinucleated telencephalic neurons, depending on the CAG repeat length [186].

Immortalized striatal precursor neurons have been derived from HD-hiPSCs that can be cultured as stable adherent cell lines and rapidly differentiated into homogeneous cultures of medium spiny neuron-like cells [190]; in agreement with earlier observations in other models [179,182], these cultures exhibited perturbed endocytosis, semaphorin and GABA receptor signaling and alterations in gene expression and metabolism [190]. These cells are as valid a tool as medium spiny neurons directly derived from HD-hiPSCs and can be used as a platform for identifying HD molecular targets and for conducting drug candidate screenings.

#### 3.5.3. Studies on the Contribution of Various Cell Types to HD Pathology

Although HD primarily affects medium spiny neurons and corticostriatal projection neurons that modulate movement and cognition, respectively, other cell types also contribute to the pathology. hiPSC-based models have been developed to explore the role of these cells. Microglia are considered to contribute to the pathology through the increased release of pro-inflammatory and oxidative stress factors and altered synaptic pruning. mHTT aggregation in developing neurons obtained from an hESC line expressing the exon1-fragment of mHTT was shown to stimulate the transcription of IL-34, a major driver of microglial expansion [181]; this response was dependent on IKKβ/mHTT interactions. Gasser and colleagues (2023) [194] showed increased phagocytic and migratory activity of microglia derived from HD-hiPSCs, compared to an isogenic control, as well as the increased release of pro-inflammatory cytokines. BBB dysfunction is found in an array of neurological disorders; Lim and co-workers (2017) [183] reported abnormal angiogenesis and barrier properties of BMECs obtained from HD-hiPSCs; BBB dysfunction was associated with altered WNT signaling, and WNT inhibition rescued the angiogenic deficits. No changes in paracellular permeability were recorded by Linville and co-workers (2022) [192] on BMECs differentiated from juvenile HD-hiPSCs, despite reduced transendothelial electrical resistance and decreased tight junction protein expression; nonetheless, these researchers identified changes in efflux activity and responses to angiogenic, oxidative and osmotic factors, as well as an augmented adhesion of immune cells in the mutant BMEC microvessels and the increased abundance of innate immune activation transcripts [192]. Aberrant interactions between the extracellular matrix and HD-hiPSC-derived BMECs were described by Hernandez and co-workers (2023) [195], associated with dysregulated integrin signaling, with the reduced adhesion of these cells to the matrix and alterations in barrier formation and function; altered transcriptomics associated with the activation of extracellular matrix and adhesion-related genes were identified both in hiPSC-derived BMECs and in hiPSC-derived astrocytes [195]. The single-nuclei RNA-sequencing of HD-hiPSC-derived astrocytes identified astrogliogenesis transcription factor dysregulation leading to maturation deficits, the inhibition of axonal guidance and altered glutamate signaling [193].

### 3.6. Amyotrophic Lateral Sclerosis Models

#### 3.6.1. Identification of Pathological Processes Associated with TDP-43 Mutations

Despite considerable genetic heterogeneity, a hallmark of ALS is the cytoplasmic mislocalization and the aggregation of TDP-43 (transactive response DNA binding protein) in MNs, which is observed in 97% of the cases. Using an sALS-hiPSC model, Keeley and colleagues (2024) [217] have demonstrated neuronal nuclear pore complex injury in ALS mediated by overactivation of the ESCRT-III nuclear surveillance pathway; this is considered to contribute to TDP-43 mislocalization and loss of nuclear function [217]. An increase in the levels of the nuclear envelope protein SUN1 has been correlated with an impaired nuclear pore complex permeability barrier in sALS-hiPSC-derived neurons; this leads in turn to increased passive nuclear influx of the ESCRT-III protein charged multivesicular body protein 7 (CHMP7); the nuclear accumulation of CHMP7 triggers nuclear pore complex injury, culminating in TDP-43 dysfunction and subsequent neuronal loss [213].

TDP-43 is involved in RNA transcription, splicing and transport processes, and reduced TDP-43 levels will affect numerous RNAs. One of these is stathmin-2, abundantly expressed in MNs and involved in neurite outgrowth and axonal regeneration. Reduced TDP-43 expression leads to premature polyadenylation and aberrant splicing in intron 1 of stathmin-2, giving rise to a non-functional mRNA molecule; its loss caused the inhibition of axonal regeneration following damage in a model of hiPSC-derived MNs [201]. The downregulation of stathmin-2 was also described in interconnected networks of mixed neuronal and glial cultures derived from hiPSCs with altered TDP-43 expression [216]; the regulation of NPTX2 by TDP-43 was demonstrated in these cultures, through binding of TDP-43 to the 3′ UTR of NPTX2. The NPTX2 protein mediates synaptogenesis and glutamate signaling via AMPA receptor clustering; the TDP-43 pathology correlated with NPTX2 accumulation, resulting in synaptic remodeling with AMPA receptor recruitment on the neuronal membrane and neurotoxicity [216].

Using hiPSCs derived from ALS patients with TDP-43 mutations, Dafinca and co-workers (2024) [214] showed an interaction between TDP-43 and ATP synthase and COX5A leading to reduced mitochondrial respiration and ATP production; mitochondrial dysfunction plays an important role in MN degeneration in ALS. Additionally, a decreased speed of retrograde mitochondrial and endosomal transport was recorded in the hiPSC-derived MNs, associated with downregulation of the motor protein complex DCTN1/dynein [214]. hiPSC-derived MNs from ALS patients carrying TDP-43 mutations showed high glutamate-induced Ca^2+^ release due to the upregulation of Ca^2+^-permeable AMPA and NMDA subunits and impaired mitochondrial Ca^2+^ uptake [202].

#### 3.6.2. Identification of Pathological Processes Associated with C9Orf72 Mutations

Increased Ca^2+^ release following depolarization was recorded in hiPSC-derived MNs from ALS patients carrying mutated C9ORF72 forms [202]; the downregulation of MICU2, a regulatory protein of the mitochondrial Ca^2+^ uniporter, and reduced expression of the calcium-binding protein calbindin, were responsible for a reduced capacity to take up cytosolic Ca^2+^ in these cells. Work by Selvaraj et al. (2018) [98] on hiPSC-derived MNs generated from ALS patients carrying mutations in the C9ORF72 gene unveiled changes in the composition of AMPA receptors that resulted in an increased permeability to Ca^2+^ and an augmented vulnerability to AMPA-induced excitotoxicity that was specific to the MNs. Such observations, initially made on hiPSC models, led to the identification of changes in the expression level of the GLUA1 AMPA subunit in ALS patients [98]. Work by Szebényi and co-workers (2021) [117] on a cerebral organoid slice model derived from ALS-hiPSCs carrying a C9ORF72 mutation demonstrated selective susceptibilities of the astrocytes and deep-layer neurons found in the organoids, with differing responses of their transcriptional, proteostasis and DNA-repair mechanisms; the accumulation of toxic poly(GA) was identified as a main contributor to DNA damage in the neurons. Studies by Maor-Nof and collaborators (2021) [205] demonstrated a key role of the transcription factor P53 in the response of MNs derived from ALS-hiPSCs carrying C9ORF72 mutations to dipeptide repeat proteins poly(proline-arginine) and poly(glycine-arginine). The stabilization of P53 due to mutated forms of C9ORF72 caused the upregulation of P53-target genes, like PUMA, leading to neurodegeneration. P53 or PUMA ablation in C9ORF72-hiPSC-derived MNs protected these cells from the toxic effects of mutant C9ORF72. In another series of studies, Guo and co-workers (2024) [215] showed the upregulation of inflammation-related pathways in MNs and astrocytes derived from C9ORF72-knockdown hiPSCs. Astrocytes derived from ALS-hiPSCs carrying mutations in VCP, SOD1 and C9ORF72 genes exhibit aberrant intron retention [206]; intron retention is a mechanism to suppress translation, and thus, the increased translation of a number of reactivity genes was observed in the mutant astrocytes, likely contributing to their entrance into a reactive state [206]. Repression by promoter methylation of a mutant form of the C9ORF72 gene, associated with fALS [126], was reported by Liu et al. (2014) [14], and it has been hypothesized that increased genome methylation may be a defense mechanism against neurodegeneration caused by the accumulation of aberrant molecular aggregates. These results are in line with the data pointing at increased global DNA methylation of ALS genomes that support the potential of epigenetics-based therapeutics for this disease [125].

#### 3.6.3. Identification of Pathological Processes Associated with FUS and SOD1 Mutations

Other models have explored the effects of mutations in other genes, such as the FUS (fused in sarcoma) RNA-binding protein gene and SOD1 (superoxide dismutase 1), also linked to ALS pathology. Both loss-of-function and gain-of-function mutations of FUS have been described. A higher expression of DNA methyltransferases, together with increased promoter methylation and reduced mRNA expression of the FUS gene have been recorded in ALS models, accompanied by the formation of cytoplasmic FUS aggregates and associated with decreased numbers of MNs in the mutant cultures [16,18]. Cytoplasmic FUS binds to 3′ UTR gene sequences and competes with other regulatory RNA binding proteins, like FMRP [19], leading to a misregulation of the expression of downstream target genes, such as HuD [203,229]. Studies on hiPSC-derived MNs obtained from ALS patients carrying gain-of-function FUS mutations demonstrated hypoexcitability and axonal transport defects linked to the reduced acetylation of α-tubulin, compared to isogenic controls; the inhibition of HDAC6, the major deacetylating enzyme of α-tubulin, rescued the defects in axonal transport [125].

Tsioras and colleagues (2023) [212] demonstrated that the accumulation of insoluble mutant SOD1 protein in an ALS-hiPSC-derived MN model interfered with the turnover of a series of proteins mainly involved in protein folding and cytoskeletal homeostasis; the valosin-containing protein (VCP), which plays a key role in ubiquitin-based degradation, accumulated with mutant SOD1 and modified its interactome, most likely affecting its role to maintain cellular homeostasis through the clearance of misfolded proteins, such as mutant SOD1. Data obtained by Günther and colleagues (2022) [208] on MNs derived from hiPSC carrying SOD1 mutations have supported the loss of mitochondrial integrity as one of the earliest pathological alterations in ALS, even prior to the accumulation of SOD1 aggregates. Namboori and co-workers (2021) [92] applied a classifier gene set of 1060 genes to separate the neurons they had obtained from ALS patient-derived hiPSC lines carrying a mutated SOD1 into distinct neural subtypes before carrying out differential gene expression analyses on these cells; as a result, these authors unveiled the activation of the TGFβ pathway in mutant MNs obtained from ALS-hiPSCs, pointing to this signaling cascade as a likely driver of gene dysregulation in ALS and MN death. Inhibition of TGFβ signaling with the small molecule SB431542 resulted in enhanced survival of these neurons. Of note, the TGFβ pathway was seen to be activated in fALS hiPSC-derived MNs and in MNs that had been microdissected from tissue from sALS patients [92]. In another series of studies, Kiskinis and co-workers (2018) [199] registered changes in neuronal excitability in MNs derived from hiPSCs carrying SOD1 mutations, compared to their isogenic controls; differences in firing action potentials run parallel to a deficit in K_V_7 currents in the mutant cells.

#### 3.6.4. Neuromuscular Culture Models

Besides organoid models, ALS-hiPSCs and their isogenic controls have been employed to establish 3D neuromuscular cultures where contractile myofibers are innervated by MNs, thus enabling functional assays [209,210]. Massih and co-workers (2023) [210] reported a decrease in muscle contraction when the innervating neurons were derived from hiPSCs generated from ALS patients with SOD1 mutations. Cheesbrough and colleagues (2023) [209] established an hiPSC-neuromuscular disease model in 96-well assay plates that allowed for imaging and functional readouts of neuromuscular transmission following optogenetic stimulation. Cultures with MNs derived from ALS-hiPSCs carrying a TDP-43 mutation evidenced weaker contractions, reduced axonal growth and reduced numbers of neuromuscular synapses and also demonstrated the use of this system as a platform to test the effect of candidate drugs [209].

#### 3.6.5. Studies on the Role of Astrocytes in ALS

A role for astrocytes in ALS pathology has been put forward. Astrocytes derived from hiPSCs obtained from ALS patients with mutated forms of C9ORF72 downregulated the secretion of antioxidant proteins and displayed increased oxidative stress and senescence; longer-term astrocyte cultures proved to be toxic to MNs via soluble factors [200]. Studies by Hall and co-workers (2017) [197] on co-cultures of MNs and astrocytes derived from ALS-hiPSC lines carrying VCP mutations identified a series of processes in MNs leading to their death and a failure of the astrocytes to support neuronal survival. Stoklund Dittlau et al. (2023) [211] reported increased reactivity, cytoplasmic FUS mislocalisation and increased secretion of inflammatory cytokines in hiPSC-derived astrocytes harboring FUS mutations compared to isogenic controls; contrary to isogenic control astrocytes, co-culture of the mutant astrocytes with neuromuscular cultures in microfluidics devices showed their failure to maintain neuronal homeostasis, with impaired MN neurite outgrowth and reduced neuromuscular junction (NMJ) formation and functionality [211]. Astrocyte-induced NMJ toxicity is regarded a general mechanism in ALS; spinal progenitors derived from sALS-hiPSCs injected into mice preferentially differentiated into astrocytes in the spinal cord and caused the loss of different neuronal populations, including the earlier loss of non-MNs (including inhibitory interneurons) and subsequent MN death, NMJ denervation and motor deficits; these processes were not observed with astrocytes differentiated from injected healthy hiPSC-derived spinal progenitors [198]. Increased levels of connexin-43 hemichannels in the membrane of astrocytes derived from sALS- and fALS-hiPSCs correlated with induced toxicity in human hiPSC-derived MNs by some factor released by the astrocytes [207]; this was not seen when culturing the MNs with control astrocytes, and blockage of the connexin 43 hemichannels conferred significant neuroprotection. Of note, toxicity to other non-MNs was also observed [207].

## 4. Stem Cell-Based Therapies for Neurodegenerative Diseases

Different types of stem cells have been investigated as novel avenues for the treatment of NDs [230,231,232]; studies and the main clinical trials from the last five years are shown in Table 2.

MSCs derived from multiple sources have been evaluated in numerous trials [299] for the treatment of multiple sclerosis (MS). However, though a degenerative disease, the primary causative pathology in MS is an autoimmune one and MSCs have long been known to have immune privilege and to be capable of protecting surrounding cells [300]. Some of the best results in stem cell trials have been achieved in spinal cord injury (SCI) [301,302,303] and macular degeneration (MD) [304,305,306]. SCI is not a classical ND, despite arising due to neuronal loss, and MD, whilst a neuronal degenerative disease, is usually treated by ophthalmic surgeons, not by neurologists. For these reasons, the focus here will be on AD, PD, HD and ALS. These diseases are also being targeted by researchers aiming to either slow degeneration or rescue the neuronal network by replacing the lost cells. With the exception of HD, a clearly defined pathological process has not been completely elucidated, and therefore, attaining a replacement of the lost cells is the main objective, even if a final halt to the disease process is not achieved.

The protection of the central nervous system by the BBB limits the access of cells and molecules that are above a certain size (around 400 Da); thus, intrathecal or target-site injection are often the only way to deliver the therapeutic cells to the site. For this reason, when attempting therapy for NDs, it is essential to distinguish between the benefits caused by the cells themselves, particularly as replacement cells in the host tissue, and some of the many beneficial effects stem cells may exert on the remaining tissues of the brain. Moreover, care must be taken regarding the therapeutical application of stem cells, as destroying the stem cell population appears to have benefits in some models of disease where an imbalance between terminally differentiated adult neuronal cells and the stem cell population may play a pathophysiological role [307]. Here, other disease-modifying treatments, including smaller molecules, like RNAi, drugs [308,309] and even the contents of exosomes derived from stem cells [310,311], might be more easily brought to the clinic. Below we discuss different approaches involving the use of different stem cell types that have been followed in the search for new therapeutics to combat such devastating diseases.

### 4.1. Alzheimer’s Disease

Until very recently, there was very little in terms of disease-modifying drugs for AD, with a few therapeutic agents that could help with symptomatology. It seems unlikely that advanced AD, with the associated loss of thousands or millions of neurons and the associated cortical structure will ever be amenable to any stem cell therapy, at least until we can image and replicate the connectome. However, the recent approval of the Aβ-targeting antibody Lecanemab, which appears to help against further progression of the disease, allows stem cell therapies to now come into their own, possibly allowing some recovery of early cellular loss in the hippocampus and neocortex [312]. This is after the disappointing results of BACE-1 enzyme inhibitors [313].

As in other neurodegenerative diseases, stem cell models are helping us understand the pathogenesis of this multifactorial illness [115,136], as well as that of Frontotemporal Dementia, the second most common dementia [314,315]. Individual hiPSC-based models are also helping develop better models for treatment, which could lead to new clinical trials [316].

#### 4.1.1. Preclinical Studies of Alzheimer’s Disease

In animal models of AD, various types of stem cells have shown potential benefits. NSCs have been shown to improve spatial learning and reduce memory dysfunction [317,318]. NSCs implanted into the hippocampus improved both memory deficits and cognitive capabilities in a murine model through the release of BDNF [319,320]. Consistent with these findings, induced NSCs (iNSCs) differentiated into neurons and glial cells and transplanted into the hippocampus of APP/PS1 mice also led to improved cognitive functions, highlighting their therapeutic potential [243]. However, despite balancing the degenerative process, NSCs do not reverse the underlying protein pathology. Interestingly, in a Down syndrome mouse model (with Down Syndrome being one of the highest risks for developing AD), NSC transplantation into the hippocampus did actually reduce Tau extracellular granules [321], possible equivalents to the NFTs which are early markers of AD.

MSCs have been extensively studied in AD and other NDs, due to their easy access and low risk. Unlike NSCs, MSCs have been shown to both reduce memory deficits and also reduce the accumulation of Aβ in AD mouse models [233,237,322]. This appears to correlate with the reduction in neuroinflammation found in other studies [235,236] with also evidence of neurogenesis and neuronal cell maturation [235,323].

Stem cell derivatives, like exosome fractions or even conditioned media, appear to possess many of the effective properties of the entire cell, not only reducing pathological protein accumulation and modulating microglial function but also reducing neuronal apoptosis [242,244,324]; as an example, Ebrahim and colleagues (2024) [242] showed the beneficial effects of MSC-derived exosomes on AD rats, through the induction of autophagy and anti-inflammatory effects, leading to improved synaptic function, memory and neurological performance; amyloid deposition and Tau accumulation were reduced in the brains of host AD rat models; moreover, improved adult neurogenesis was registered. In a 5-fAD mutation (5XFAD) mouse, the intranasal administration of small extracellular vesicles (EVs) derived from hMSCs led to a significant improvement in cognitive tests and a reduced Aβ plaque load in the hippocampus, slowing down the progression of the disease [234]. Comparatively, the intravenous administration of iNSC-derived EVs in 5XFAD mice resulted in significant cognitive improvements, reduced Aβ levels, decreased phosphorylated Tau propagation, and enhanced dendritic spine density in the prefrontal cortex and hippocampus of these animals [239]. In another study, while the application of NSC-derived exosomes helped in preserving mitochondrial function, suppressed astrocyte activation and resulted in improved cognitive performance in a murine model of AD, no significant effect on Aβ deposition was found [325]. The directed engineering of these exosomal components can further enhance their positive effects [326]. One more adult stem cell type with a proclivity to produce neuronal cells due to its neural-crest derivation is the dental pulp-derived stem cell, studied in AD and other conditions [327].

Studies investigating hematopoietic stem cell (HSC) transplantation show promising results for AD treatment, modulating neuroinflammation and cognitive function, as well as reducing amyloid plaques in 5XFAD mice [240]. One study found that the intra-CNS transplantation of HSCs engineered to overexpress TREM2 reduced Aβ aggregation, decreased neuroinflammation and improved memory in 5XFAD mice [245]. Similarly, mutant TREM2-AD mice showed restored microglial function, preventing neurodegeneration after replacing mutant microglia throughout the brain with circulation-derived myeloid cells following systemic blood stem cell transplantation [241].

iPSCs have revolutionized stem cell research and are also being used in animal model research in AD. As an example, protein-induced iPSCs derived from mouse fibroblasts reduced amyloid plaques and improved cognition in a 5XFAD transgenic AD mouse model; differentiated into glial cells, they also showed the upregulation of oligodendrocyte genes [328]. hiPSC-derived neuronal precursors implanted in the hippocampus developed into mature cholinergic neurons, producing a functional benefit [329]. Interestingly, iPSCs can also be used to produce non-neural cells, such as pericytes [330], which have been linked to increased capillary constriction in AD [331], and also macrophages that have been transduced to produce Neprilysin-2, which can degrade Aβ.

NPC transplantation has also shown some positive results in animal models of AD [332]. An Australian study reported on a treatment using skin-derived NPCs being microinjected into the hippocampus via MRI-guided stereotaxis. A canine cognitive dysfunction model was used that resembles mild AD dementia-like syndrome. Out of five dogs which completed the trial, two amazingly had the full reversal of the syndrome lasting up to 2 years, whilst the others improved very significantly [333]. As in comparative oncology for cancer, understanding species and disease differences in the relevant AD-like illnesses will no doubt help in human understanding and possible treatment development.

As seen with other NDs, combinatorial therapies with stem cells combined with trophic factors, like NGF, can bring about the best results [334]. Apart from growth factors, other drug–cell combinations may prove useful, enhancing cellular functions or the lifespan [335,336,337]. Exosomes may provide an easier way into trials; being cell-free and potentially allogeneically developed, they could be considered relatively “off the shelf” [338]. In the future, methods to differentiate other cell types *in vivo* and replace the neurons lost in NDs may add to the arsenal of available treatments [339].

#### 4.1.2. Clinical Trials for Alzheimer’s Disease

Clinical trials for AD are being developed but are relatively in their infancy. One trial, which included a number of neuronal diseases’ response to fat-derived autograft injections, showed some slowing down of AD in a small number of patients [246]. Building on these findings, a new phase I, open-label safety study (NCT05667649) is evaluating escalating doses of autologous, ex vivo-expanded adipose-derived stem cells (RB-ADSCs) in patients with mild-to-moderate AD [253]. Similarly, in a phase II clinical trial in USA, AD patients received an intravenous infusion of an autologous adipose-tissue-derived MSCs (NCT04482413) [252].

Another phase I safety trial implanted cord-blood-derived allogenic MSCs into the lateral ventricle with some adverse effects in mild-to-moderate AD patients [247]. Another phase I trial using a low dose of a commercially available allogenic MSC preparation derived from bone marrow cells showed achievement of the safety endpoint and results suggestive of improved neurocognition [248]. Other ongoing clinical trials are also evaluating MSC-based therapies. A phase II study is assessing the safety and tolerability of intravenously administered allogeneic human MSCs in subjects with mild-to-moderate dementia due to AD (NCT02833792) [251].

Exosome therapies, which avoid the tumorigenic risks of stem cell transplantation, show promise, although challenges in production scalability and targeting precision remain. The safety of allogenic adipose MSC-secreted exosomes intranasally administered in patients with mild-to-moderate AD has been evaluated in a phase I/II clinical trial [249,250]. It is hoped that combinations of different stem cells and factors (even secreted by these same cells) will help progress the treatment for this difficult condition in the future [238].

### 4.2. Parkinson’s Disease

Like in most diseases, stem cells, particularly iPSCs, are playing a major role in discovering the pathophysiological processes behind NDs, such as the effect an impaired BBB—that in certain cases may be due to inflammation—may have [97]. They are also playing a significant role in drug development [128]. In most cases of PD, the exact cause of the loss of dopamine-producing neurons in the substantia nigra is unknown, and therefore, the development of stem cell therapies has been actively pursued.

#### 4.2.1. Preclinical Studies of Parkinson’s Disease

ESCs, including PESCs or parthogenetic ESCs (cells derived from embryonic-like structures from activated unfertilized oocytes), iPSCs and NSCs are all being evaluated for differentiation into DA neurons [340]. Preclinical research assessed the tumorigenicity risk and biodistribution of ESCs and ESC-derived DA neurons implanted in non-human primates [341,342]. As an example, López-Ornelas et al. (2023) [260] transplanted hESC-derived DA neurons into Parkinsonian non-human primates, demonstrating partial motor recovery and long-term dopamine release. Imaging techniques revealed graft survival and axonal density improvements, suggesting the robust integration of transplanted cells. On the other hand, Takagi et al. (2005) [343] demonstrated functional improvement after implanting primate ESCs into PD patients.

iPSCs can be more naturally differentiated into DA neurons using miRNA [261] and even scaffolding systems [344]. Novel methods are also providing better markers to predict the prognosis from differentiated DA cells [258]. Hallett et al. (2015) [345] demonstrated the potential of autologous hiPSC-derived DA neurons for PD. In a Parkinsonian monkey model, the long-term survival of transplanted neurons was observed, with motor improvements and extensive outgrowth into the putamen. Notably, these effects were achieved without immunosuppression, supporting the feasibility of autologous hiPSC therapy for PD. Daadi and colleagues (2024) [263] further developed hiPSC-derived DA neurons, integrating physical and cognitive training to enhance graft integration and functional recovery in a nonhuman primate model of PD. Human 3D-cultured midbrain organoids derived from hiPSCs have also been transplanted as tissue pieces into the striatum of 6-hydroxydopamine (6-OHDA) mice, a PD mouse model, showing robust survival and maturation and resulting in reversed motor deficits, as well as the establishment of bidirectional neural connections, while avoiding tumor formation [262].

hNSCs implanted into the SVZ differentiated into DA neurons in a rat PD model and resulted in the relief of some of the symptoms [346,347]. Similar beneficial results were seen in rodent and primate models using MSCs [257,348]. Moreover, exosomes derived from MSCs have been reported to promote angiogenesis and neuronal recovery, alleviating DA neuron damage and motor deficits in PD mice [254,256]. MSCs have the additional benefits of being less likely to produce tumors and may often be easily derived from the patient (e.g., from fat tissue) [349]. This allows them to be easily acquired even for repeat infusions/implantations [272], something that may be necessary in these chronic degenerative diseases. Other methods of the delivery of such cells have been attempted, such as vascular injection into the artery supplying the substantia nigra [350]; even intravenous delivery could improve outcomes [259]. Another multipotent (as opposed to pluripotent) stem cell that may be of use in the future is the dental pulp-derived stem cell [351].

The co-delivery of growth factors (even through stem cell modification) or co-administration of protective factors, including polyphenols, may recreate the optimum situation for DA neuron survival [352,353]. An example is the long-term effects of HSC-based glial derived neurotrophic factor (GDNF) via exosome transfer and direct diffusion that improved motor function for up to 12 months, preserved DA neurons and selectively targeted degenerative areas in the brain of 1-methyl-4-phenyl-1,2,3,6-tetrahydropyridine (MPTP) PD mice [264]. In line with this, Narashiman et al. (2024) [265] presented the use of cryogel microcarriers loaded with GDNF to enhance DA graft survival and reinnervation in Parkinsonian rat brains. The study demonstrated increased graft viability and improved fiber sprouting, paving the way for optimized cell transplantation strategies.

#### 4.2.2. Clinical Trials for Parkinson’s Disease

Few clinical trials have published some results in the field of stem cell therapies for PD. By 2024, a number of cell therapies were in phase I clinical trials, few in phase II and none in phase III. Numerous drugs are still targeting symptomatic relief, including LY03003 and Opicapone, in phase III trials, whilst a few target α-syn as part of the pathogenetic process, like Memantine and Citalopram [354]. It is likely that similarly to other NDs, a combination of disease-modifying drugs to slow progression and stem cells to replace lost cells and further reduce cellular loss will provide the ultimate treatments. Other approaches, like deep brain stimulation, are already helping PD patients [355].

In 2010, bone marrow-derived MSCs injected into the lateral ventricle wall resulted in some improvements in patients in the Unified Parkinson’s Disease Rating Scale (UPDRS) [356]. The nature of the trial did not allow for a clear quantification of the benefit, though safety and a lack of side effects were noted. However, a concomitant trial with the intraarterial injection of MSCs resulted in a significant improvement in disability, activities of daily living, depression and quality of life [350]. Trials using cells derived from the fetal retinal pigmented epithelium also gave considerable improvements (UPDRS), but these peaked at 12 months after use and then regressed [357], possibly due to the immune rejection of autologous-derived cells.

A more recent clinical trial used stereotactic surgery to implant allogeneic cells from a fetal-derived NPC line into the putamens of eight patients who were also given cyclosporine from 10 days prior to surgery until 1 month afterwards. There was no evidence of immune rejection of the tissue and biological evidence of functional graft activity was still being detected up to 4 years later [358]. In six of the seven patients who could be followed up, a residual improvement in their UPDRS scores remained at 4 years, especially in the OFF state.

One of the most interesting recent clinical results was one which showed sustained clinical improvements in the PD symptoms of two patients, following injection into accessible sites in the face of the non-modified autologous fat-derived stromal vascular fraction (SVF), which is what normally contains the adipose MSCs [359]. These injection sites should allow for relatively easy access to the cerebral circulation allowing the secretome of these cells to influence the brain tissues. In another study, Zhuo and colleagues (2024) [277] evaluated the effects of hypoxia-preconditioned olfactory mucosa-derived MSCs on PD models and patients. By enhancing microglial regulation and mitochondrial recovery, these cells promoted neuronal survival and reduced neuroinflammation.

Sometimes, whilst an improvement is hard to come by, preventing deterioration may be the next best target. In five patients with progressive supranuclear palsy, a rare and progressive form of PD with no treatment options, autologous bone marrow-derived MSC injection into the cerebral arteries stabilized the condition for at least 6 months [360].

Another phase I/II clinical trial investigated allogeneic MSCs differentiated into NSCs to assess their safety and efficacy in PD [267]. More recently, Jiang and colleagues (2024) [275] investigated the intranasal transplantation of ANGE-S003 NSCs in a phase I clinical trial for PD. The study found significant improvements in motor symptoms, with peak efficacy observed at six months post-treatment.

Fetal brain-derived tissue implantation in PD patients was performed a few years ago with short-term benefits [361]. More recently, Kirkerby et al. (2023) [362] demonstrated the safety and efficacy of STEM-PD, a hESC-derived DA progenitor product, in preclinical PD models. The associated trial (NCT05635409) is an early-phase study assessing the safety and feasibility of intraputamenal STEM-PD transplantation in moderate PD patients [274], paving the way for clinical applications. Park and colleagues (2024) [276] also described a scalable method to derive midbrain DA progenitors from hESCs. Their findings supported a minimal effective cell dosage for transplantation, culminating in the approval of a phase I/IIa clinical trial in Korea [276]. Also, a phase I trial recently completed (NCT04802733) evaluated Bemdaneprocel (BRT-DA01) [270], using dopamine-producing neurons derived from hESCs [269]. This therapy demonstrated long-term safety, tolerability and therapeutic potential in alleviating motor symptoms in PD patients. The promising results supported advancing to phase II trials to further assess efficacy in a larger population (NCT05897957) [271].

On the other hand, hiPSCs are a cell source that can be derived from patients themselves and, which due to their autologous derivation, are unlikely to induce immune responses, unlike heterologous ESC-derived cells. On this basis, a number of hiPSC-derived clinical trials are in course [363]. A groundbreaking study revealed that a PD patient who underwent the autologous transplantation of hiPSC-derived midbrain DA progenitors experienced either stabilization or an improvement in clinical symptoms 24 months after the procedure [266]. Though easily sourced, their derivation is somewhat time-consuming and complex, limiting widespread usage. Their importance in disease modeling and drug development in PD and other NDs continues to increase [364]. Fears of tumorigenesis seem to have been largely laid at rest by pre-clinical safety and efficacy trials, which have even shown functional benefits [255].

### 4.3. Huntington’s Disease

Being an autosomal dominant genetic disease, HD is more amenable to possible non-cellular genetic and protein-based disease-modifying therapies than other NDs. The aim in many of these is to ablate the effect of the toxic dominant negative mHTT protein.

#### 4.3.1. Preclinical Studies of Huntington’s Disease

MSC-like stem cells (including some modified to secrete neurotrophic factors, like BDNF) and even NSCs have all had significant benefits in a number of animal models of HD [94,365]. Moreover, one study showed that the efficacy of bone marrow-derived MSCs is dependent on their passage number, particularly noting that high-passage cells exhibited better survival and greater neuroprotective effects (likely due to the upregulation of neurotrophic factors, like BDNF) when transplanted intrastriatally in a mouse model of HD [366]. In relation to NSCs, the transplantation of a conditionally immortalized NSC line in a quinolinic acid-lesioned rodent model of HD showed substantial behavioral improvements, neuroinflammation reductions and successful differentiation into medium spiny neurons, the cell type most affected in HD. These therapies both prevent the further loss of neuronal cells [367,368] and regaining motor function [369]; genetically corrected iPSCs can also be differentiated into MNs [370]. As an example, Cho and colleagues (2019) [278] explored the combined efficacy of stem cell and gene modification therapy to treat HD by using NPCs derived from Rhesus monkey iPSCs, genetically modified to reduce mHTT levels. Transplantation into HD mice improved motor functions, increased their lifespan and promoted differentiation into GABAergic neurons [278].

Cell therapies using the transplantation of hESCs labeled with superparamagnetic iron oxide nanoparticles have further shown promising results in a rat model of HD. The outcomes indicated significant motor improvements, cell migration to degenerated brain areas and successful differentiation into GABAergic neurons, as observed through behavioral testing and MRI monitoring [280].

#### 4.3.2. Clinical Trials for Huntington’s Disease

Phase I and II clinical trials have been run based on a preclinical study of intravenously administered MSCs derived from dental pulp in a rat model of HD [281]; the study yielded promising results, such as increased levels of neurotrophic factors and promoted neuroprotection and neurogenesis in the striatum and cortex. The clinical trials demonstrated potential improvements in motor functions with low doses of a product (NestaCell^®^) based on these cells expressing high levels of BDNF in HD patients [282,283,284]. Another ongoing phase III clinical trial has also assessed the efficacy of this therapy (NCT06097780) [285].

Whilst many of the above-mentioned therapeutics are essential to reduce/abrogate the effect of the recognized toxic proteins, which are causative of neuronal degeneration in HD, recovery in an adult human brain may not be sufficient to correct functional deficits caused by the loss of the GABAergic inhibitory spiny neurons of the forebrain striatum. Ideally, the integration of these cells into the host brain circuity, which requires human clinical trials, will most probably benefit from an enhanced environment and exercise, driving this neurocircuitry to reform in its most functional form [371]. We believe that human stem cell trials in HD patients will kick off in force when non-stem cell therapeutic disease-modifying treatments produce considerable success and succeed at entering the marketplace.

### 4.4. Amyotrophic Lateral Sclerosis

ALS (otherwise known as Lou Gehrig’s disease) is one of a family of MN diseases, most of which have an unclear etiology and inappropriate treatment options leading to rapid death. Most disease-modifying drugs are either in trials or have only been shown to have minimal effects on survival such as a 3-month extension of life [372].

#### 4.4.1. Preclinical Studies of Amyotrophic Lateral Sclerosis

Whilst success with stem cell therapies may still be somewhat distant, stem-cell derived model systems are of extreme benefit in enhancing the understanding of the various types of the condition [117,293,373]. Very interestingly, these models are also helping the development of potentially very useful pharmacological [374], as well as non-pharmacological, treatments [375]. In a rat model of mSOD1-caused ALS, the implantation of an iNPC line transduced with GDNF provided neuroprotection and enhanced MN survival [289].

Considerable research with MSCs has shown their neuroprotective capabilities in various model systems [376,377]. In line with this, the therapeutic potential of MSC-derived EVs, focusing on their neuroprotective and immunomodulatory properties, has also been explored both *in vitro* and in ALS mouse models [288,378]. More precisely, Giunti and colleagues (2021) [288] identified specific miRNAs in MSC-derived small EVs that modulated microglial activity by suppressing pro-inflammatory markers via the p38 MAPK pathway. *In vivo*, these MSC-derived EVs reduced neuroinflammation markers in the spinal cord of EAE-affected mice, although it had limited impact on disease progression [288].

In another study, the intravenous administration of Multilineage-differentiating stress-enduring (Muse) cells, which are endogenous pluripotent-like stem cells, demonstrated selective homing to damaged tissues in G93A-transgenic ALS mice. This resulted in their targeted migration to the lumbar spinal cord, differentiation into glia-like cells expressing GFAP and significant motor function improvements, including enhanced rotarod performance, muscle strength and reduced myofiber atrophy [287].

The transplantation of iPSCs may be an effective treatment to improve the MN environment [379]. The therapeutic potential of neural progenitors derived from iPSCs has also been addressed in a transgenic SOD1^G93A^ rat model of ALS, where intraspinal transplantation preserved MNs, slowed disease progression and extended survival [286]. These findings further support the neuroprotective effects of iPSCs and their ability to regulate the spinal microenvironment, offering promise for ALS treatment.

#### 4.4.2. Clinical Trials for Amyotrophic Lateral Sclerosis

Like with PD, initial trials with fetal-derived tissue (in this case spinal cord-derived NPCs) in ALS patients’ spinal cords were started as far back as 2010 [380,381]. A clinical trial in Spain on 11 patients showed evidence of increased numbers of MNs in each of the treated (as opposed to untreated) spinal segments when autologous bone marrow mononuclear cells (which may have included hematopoietic and possibly mesenchymal stem cells) were infused into posterior spinal funiculi [382]. MNs in these segments showed less degenerative ubiquitination and were surrounded by CD90^+^ cells, indicating that the stem cell component from the bone marrow infusion may have migrated towards the MNs. These results led to a phase II clinical trial (NCT04849065) [297].

Phase I trials with allogenic fetal-derived or umbilical cord-derived NSCs and MSCs have also shown temporary improvements in functional scores [383,384]. Early phase I trials attempted to use a number of different cell types but were unable to pinpoint the ideal cell to progress with to later clinical trials [385].

A rather recent phase II clinical trial with the repeated intrathecal injections of autologous MSCs in ALS patient showed them to be safe and well-tolerated. The treatment reduced the rate of disease progression in a significant portion of patients, with some even experiencing functional improvements [292]. The effect of the intrathecal administration of Wharton’s Jelly MSCs on the immune system of ALS patients has also been addressed in another phase I/II clinical trial (NCT04651855) [295]. Also, a phase III ALS trial used MSCs induced to secrete high amounts of neurotrophic factors. Patients were assessed by means of the ALS Functional Rating Scale (ALSFRS-R). Despite no statistical significance between the treated and placebo group, clear positive trends were noted [386]. Most interestingly, a trial using repeated doses of Neuronata-R^®^ (lenzumestrocel), an autologous bone-marrow-derived MSC product has very recently been published, showing significant increases in survival in ALS patients in Korea [294]. Another promising clinical trial evaluated the efficacy of autologous bone-marrow-derived MSCs, induced to secrete NTFs (NurOwn^®^). A higher proportion of treated participants showed increased neurotrophic factor levels and decreased inflammation, as well as ALSFRS-R improvement, compared to the placebo [290,291]. Although direct microinjection into the dorsal horn of the spinal cord did not appear to improve the therapy, the risk of side effects must be considered. A more recent study showed benign neuromas close to the injection site, at post-mortem stages, which might have grown much larger and caused pressure effects had the patient lived longer [387].

Drug-repurposing efforts based on iPSC-derived models led to the identification of bosutinib, a Src/c-Abl inhibitor, as a candidate therapy for ALS. Following encouraging phase I safety data, a phase II trial is ongoing, investigating the drug’s long-term efficacy in slowing disease progression, as shown by measurements based on ALSFRS-R scores [296].

Another ongoing phase I trial is evaluating treatment safety and tolerability of human glial restricted progenitor cells (hGRPs; Q-Cells^®^) transplantation in ALS, with each subject receiving a single transplantation of Q-Cells^®^ into either the lumbar or cervical spinal cord (NCT02478450) [298]. Together, these efforts illustrate the multifaceted potential of stem cell therapies while underscoring the need for rigorous, large-scale trials.

## 5. Discussion

**Figure 1:**
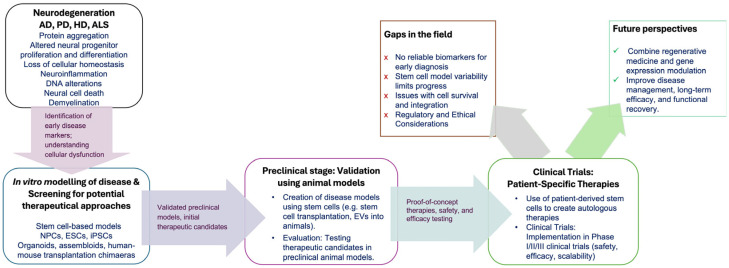
Flowchart summarizing the importance of stem cells in basic research and in possible clinical applications for ND.

For a long time now, most societies in the developed world have seen their life expectancy become extended. However longer living is not being wholly translated into an extension of healthy life expectancy, with the incidence of diseases, such as cancer and NDs, on the rise [388]. No cure exists for devastating NDs, like AD, PD, HD and ALS; thus, there is a dire need to identify the molecular mechanisms leading to ND onset and progression, so that clinical targets may be established and new efficacious therapeutics developed. Stem cell-based approaches have become key players in these studies, as summarized in Figure 1.

### 5.1. Stem Cell-Based Models for Early Diagnosis

It has been shown that common pathological hallmarks—aberrant protein aggregation, impaired proteostasis and mitochondrial dysfunction—often emerge long before clinical symptoms become apparent. Among them, there is an impairment in adult hippocampal neurogenesis that often correlates with non-motor deficits, like anxiety, depression and memory loss. Nonetheless, there are no reliable markers to date that allow for an early diagnosis of these patients; diagnosis by the time overt symptoms have made an appearance makes it impossible to succeed at slowing down or halting disease progression, let alone reversing the damage already suffered. In this context, stem cell-based models have emerged as indispensable tools for recapitulating early pathological events and for validating candidate biomarkers, such as circulating microRNAs, exosomal proteins and altered metabolite profiles. New technological advancements are needed that enable the identification of early changes, preferably in a non-invasive manner [389]. The application of omics and high-throughput genetic analyses, together with high-content imaging and artificial intelligent systems, should support these efforts.

### 5.2. Advances in Preclinical Models and Translational Approaches

The translation of preclinical findings into successful clinical outcomes remains a major challenge, and thus, reliable models of disease must be developed that allow us to gain information beyond the precious data already obtained from patients’ tissue samples and animal models. Approaches, such as optogenetic modulation to induce protein aggregation in the cultures, are helping to produce models that more closely reproduce the human pathology and that may be used to screen for therapeutic candidates [128,390].

Additionally, advances in bioengineering—such as the bioprinting of three-dimensional structures that faithfully mimic native tissue (with the incorporation of differentiation and survival factors into bioinks) [391,392]—are enabling the creation of more physiologically relevant *in vitro* systems. Additional models, including assembloids [91] and organ-on-a-chip cultures [393], facilitate the study of neural cells in their native microenvironment and in their interactions with other tissues. Multiomics analyses and drug testing on these models will undoubtedly provide new clinical targets and promising drug-leads. Very importantly, recent changes in the US FDA regulations now allow for clinical trials to be conducted without prior animal testing [394]. hiPSC-derived cultures are becoming models of choice, and open-access hiPSC biobanks are now available [395]. Despite these advances, challenges persist in translating preclinical findings into clinical success. Moreover, although the concerns about a possible tumorigenicity of implanted hiPSCs are waning, these cannot be obviated; also, very controlled differentiation protocols need to be established prior to their consideration as exogenous cells for treatment. Integrating hiPSCs with omics technologies and patient-derived data may improve preclinical models and enable more personalized treatments for NDs.

### 5.3. Stem Cell Therapies and Combinatorial Therapeutic Approaches

Regarding the use of stem cells in transplantation therapy, it will be necessary first to consider the degree of damage already suffered by the patient; again, an early diagnosis will be of the utmost importance. Some good results are coming from NPCs and, especially, MSCs, which have already been used in the clinic for a long time. Despite these advances, challenges persist in translating preclinical findings into clinical success. Key limitations include achieving adequate cell survival and post-transplantation integration, immune rejection, scalability, ethical concerns and the complexity of disease mechanisms.

Stem cell therapies can be significantly enhanced through combinatorial approaches. Gene-editing technologies, such as CRISPR/Cas9, are being applied to correct pathogenic mutations in patient-derived iPSCs, enabling the development of autologous cell therapies with reduced immune rejection. Viral-vector-mediated gene delivery, when combined with stem cell transplantation, enhances neuronal survival and functional recovery by promoting the expression of neuroprotective factors [396]. Additionally, the integration of stem cell therapy and antisense oligonucleotides (ASOs) provide a powerful tool for gene expression, particularly in diseases driven by toxic protein accumulation, such as HD. In ALS, ASOs have shown promising results in MNs derived from patient-specific iPSCs, which serve as valuable models for screening, optimizing efficacy and predicting patient responses, accelerating their clinical translation [397].

Small molecules targeting neurogenic pathways are being tested on stem cell-derived organoids to identify compounds that stimulate neuronal differentiation and synaptic connectivity. This approach not only aids in drug discovery but also informs strategies for enhancing the regenerative potential of transplanted neural progenitor cells. These synergistic strategies aim to address both cellular loss and the underlying molecular mechanisms driving disease progression [398].

### 5.4. Future Directions

The future of stem cell therapies lies in integrating multiple approaches into a unified treatment strategy. Developing integrated therapeutic strategies that combine regenerative medicine with targeted gene modulation is critical for addressing the multifactorial nature of NDs. It will be very important to consider the differences observed in the way the various stem cell niches behave in NDs (e.g., there may be altered hippocampal neurogenesis while no changes occur in the SVZ niche), as well as those cases in which increased numbers of stem cells may not be the solution but a source for greater damage. Of course, stem cell therapy may not only come from implanting the cells themselves but also from exploiting their secretome, their capacity to release factors (some of these may be contained in exosomes) that may act as disease-modulators and help slowing down disease progression. Exosome therapies, which avoid the tumorigenic risks of stem cell transplantation, show promise [399]; for example, combining exosomes with Aβ-targeting therapies could enhance efficacy in AD, although challenges in production scalability and targeting precision remain.

It is likely that the treatment of NDs like HD come from the hand of genetic or pharmacological therapy; the synergy between stem cell therapies and pharmacological strategies could advance beyond current symptomatic treatments by addressing underlying disease mechanisms. Also in these cases, stem cell-based models will likely play a key role, aiding in the development and testing of promising therapies.

## Figures and Tables

**Table 1:** ND studies conducted on stem cell-based models.

Disease	Reference	Stem-Cell-Based Model	Main Outcomes
AD	Choi et al. (2014) [107]	3D cultures of neurons and glia differentiated from fAPP- or APP/PS1 hNPCs	Aβ accumulation leads to increased levels of p-Tau and Tau aggregation. GSK3β regulates Aβ-mediated Tau phosphorylation. Dramatic increase in 4R-Tau in 3D cultures *vs.* 2D cultures.
	Murator et al. (2014) [149]	APP-hiPSC-derived neurons	Aβ accumulation precedes AD-associated tauopathy.
	Hossini et al. (2015) [114]	sAD-hiPSC-derived neurons	Characterization of the neuronal transcriptomes. Establishment of AD-related protein association networks.
	Espuny-Camacho et al. (2017) [143]	Cortical precursor cells derived from non-affected hiPSCs and neurons derived from miPSCs transplanted into the brains of an AD mouse model containing Aβ plaques	Human neurons respond to Aβ pathology differently than their murine counterparts *in vivo*. Transplanted human neurons show signs of neurodegeneration that are not observed with the murine neurons.
	Kondo et al. (2017) [116]	Highly pure cultures of cortical neurons derived from AD- and healthy control-hiPSCs	Screening of a pharmaceutical library and validation of compounds that improve Aβ phenotypes.
	Kimura et al. (2018) [150]	Neurons derived from AD- and healthy control-hiPSCs	Beneficial effect of nobiletin, with reduced Aβ accumulation; effect possibly mediated by increased neprylisin expression.
	Park et al. (2018) [118]	3D co-cultures of an adult microglial cell line with neuron/astrocyte bicultures derived from APP- and APP-hiPSC-NPCs	Microglial recruitment, secretion of proinflammatory cytokines and chemokines and neuron/astrocyte loss. Physical interactions between neurons and microglia, resulting in cleaved axons and neurite retraction.
	Ghatak et al. (2019) [119]	Neurons and cerebral organoids derived from APP or PS1-hiPSC and from isogenic hiPSC controls	Increased excitatory and decreased inhibitory synaptic activity, decreased neurite length and branching, increased basal presynaptic vesicle release and increased sodium current density in AD-neurons. Accelerated maturation of neurons carrying a PS1 mutation.
	Konttinen et al. (2019) [151]	Microglia-like cells differentiated from hiPSCs obtained from healthy controls, from AD patients with various genetic backgrounds and from their isogenic controls	Profound effect of APOE4 on microglial function (altered phagocytosis, migration, metabolic activity and cytokine secretion).
	Meyer et al. (2019) [152]	NPCs derived from sAD-hiPSCs and from APOE4 gene-edited and control hiPSCs	Reduced NPC proliferation, premature neuronal differentiation and reduced NPC renewal in AD- and APOE4- cultures. Accelerated synapse formation and increased electrical excitability. REST dysfunction and epigenetic dysregulation.
	Shin et al. (2019) [100]	Cerebrovascular model: 3D microfluidic platform containing neurons and glia differentiated from control, APP- or APP/PS1-hNPCs and human brain endothelial cells	Increased BBB permeability, higher ROS levels and IFNγ secretion and Aβ accumulation at the vascular endothelium in AD cultures.
	McQuade et al. (2020) [153]	*In vitro* analyses and transplantation into AD mouse brain of TREM2-hiPSC-derived microglia	Altered transcriptome, greater susceptibility to stress and functional impairment.
	Najm et al. (2020) [144]	APOE4-hiPSC-derived neurons in human–mouse chimeras (*vs.* APOE3-hiPSC-derived cultures)	Effect of APOE4 expression on neuronal and microglial responses to Aβ aggregate formation. Greater susceptibility of APOE4-inhibitory neurons, compared to APOE4-excitatory neurons.
	Zhao et al. (2020) [154]	Cerebral organoids from APOE3-hiPSC or APOE4-hiPSC from individuals with normal cognition or AD dementia	Greater apoptosis and decreased synaptic integrity in APOE4-organoids from AD patients; increased stress granule formation and higher mRNA levels of mature neuronal markers. APOE4 increases Aβ and exacerbates Tau pathology.
	Bassil et al. (2021) [95]	hiPSC-derived neurons and microglia, co-cultured with primary astrocytes and exposed to exoge-nously added synthetic Aβ42 oligomers	Aβ plaques, synapse loss, axon fragmentation, dendritic atrophy, p-Tau induction and neuronal cell death. Microglia internalization of Aβ42 is critical for amyloid plaque formation. Microglial neuroprotection is lost in a neuroinflammatory state. Neurotoxicity blocked by co-treatment with an anti-Aβ antibody.
	Guttikonda et al. (2021) [96]	Tri-cultures system with neurons, astrocytes and microglia. Neurons derived from APP-hiPSC and from isogenic hiPSCs	Increased neuroinflammatory responses in APP model, mediated by microglia–astrocyte signaling.
	Lagomarsino et al. (2021) [2]	Neurogenin-2-mediated induction of neuronal differentiation of hiPSC lines from 53 donors, including 16 individuals diagnosed with late-onset AD (genome sequencing, cognitive scores and quantitative neuropathology available from each donor)	RNAseq and proteomic profiling of hiPSC-derived neurons. Identification of proteins and networks associated with AD phenotypes and validation of relevant associations in brain.
	Jorfi et al. (2023) [155]	3D neuroimmune microfluidics model: co-culture of fAD-hNPC-derived neurons and astrocytes with hiPSC-derived microglia	Aβ deposition and Tau pathology in AD-neuron–glia co-cultures. Activated microglia in co-cultures with neurons and astrocytes carrying APP and PS1 mutations (*vs.* controls). CD8+ T cells and microglia synergistically promote neuronal and astrocyte damage.
	Liu et al. (2023) [156]	APOE4-hiPSC-derived microglia	Impaired microglial responses to limit the development of amyloid pathology.
	Nakatsu et al. (2023) [83]	18 hiPSC lines derived from healthy controls and from sAD and fAD patients	Alterations in the differentiation of AD-hiPSCs when senescence is induced.
	Watanabe et al. (2023) [157]	Co-culture of primary mouse neurons with astrocytes derived from APOE4-hiPSCs (*vs.* APOE3-hiPSCs)	Synaptic dysfunction in co-cultures with APOE4-astrocytes.
	Choe et al. (2024) [138]	AD-hiPSC-derived cerebral organoids	APP and PS1 mutations lead to Aβ accumulation, tauopathy and increased apoptosis.
	Ding et al. (2024) [158]	Microvascular endothelial cells and brain pericytes derived from hiPSCs with different APOE genotypes	APOE4: exacerbated amyloid clearance and deposition at the model BBB, contrary to APOE2.
	Mancuso et al. (2024) [148]	Microglia derived from control, TREM2- or APOE-modified hiPSCs engrafted into the brains of WT or APP-mice	Differences in the response of human microglia to Aβ pathology, compared to murine microglia. Differential effect of TREM2 and APOE on the response.
	McDiarmid et al. (2024) [159]	SORL1-hiPSC-derived NPCs	Phenotypic drug screen identifies 16 small molecules that reverse the mutant phenotype.
	Parra Bravo et al. (2024) [127]	Neurons derived from hiPSCs engineered to express 4R Tau and 4R Tau carrying the P301S MAPT mutation	Identification of over 500 genetic modifiers of seeding-induced Tau propagation. Identification of the UFMylation cascade as a novel key modifier. Neuronal activity and endolysosome and retromer dysfunction enhance Tau propagation.
	Penney et al. (2024) [160]	Microglia derived from TREM2-hiPSCs and from isogenic hiPSCs	Proinflammatory gene expression signature and enhanced response to proinflammatory stimuli of mutant microglia. Impaired injury response and clearance abilities *in vitro* and increased pruning activity *in vivo*.
	Qu et al. (2024) [146]	NPCs derived from APP-hiPSCs or isogenic controls transplanted into mouse brain	Amyloid pathology, impaired neurite outgrowth and dysregulated transcriptional and metabolic signatures of mutant neurons.
	Salcedo et al. (2024) [161]	APP- or PS1-hiPSC-derived astrocytes and neurons	Increased glycolysis and glucose oxidative metabolism and elevated glutamate synthesis in mutant cells. Reduced expression of the excitatory amino acid transporter 2 in astrocytes. Hypermetabolic astrocytes, possibly linked to toxic glutamate accumulation.
	Saurat et al. (2024) [122]	Highly pure cultures of cortical neurons differentiated from APP-hiPSC and iso-genic control	Neddylation inhibition induces cellular aging and synergizes with AD genetic risk to drive Tau pathology and neuronal death.
	Siew et al. (2024) [162]	hiPSC-derived microglia	pTau-induced release of Gal3 from microglia. Gal3 increases the accumulation of pathogenic Tau.
	Targa Dias Anastacio et al. (2024) [93]	fAD-hiPSCs and iso-genic controls and healthy controls. PS1-fAD-hiPSC-derived neurons	Altered Ca^2+^ responses to glutamate and AMPA in PS1-neurons precede amyloid and Tau phenotypes.
	Pavlou et al. (2025) [108]	3D model of the human neurovascular unit: primary brain endothelial cells and pericytes co-cultured with fAD-NPC-derived neurons and astrocytes	Aβ plaques in neuronal and vascular compartments, increased BBB permeability, dysregulation of endothelial and pericyte markers and morphological alterations in the vascular networks, reproducing AD pathologies.
	Rao et al. (2025) [163]	Neurons derived from APOE4, isogenic APOE3 and APOE-KO hiPSCs transplanted into the hippocampus of hAPOE4 or hAPOE3 knock-in mice	Possible role of neuronal APOE in modulating microglial status. Microglia promote human neuronal APOE-related Aβ and Tau pathologies.
PD	Wang et al. (2012) [106]	PD-adult NPC-derived neurons and astrocytes. PD-adult NPCs trans-planted into murine brains	Alterations in the ability of NPCs to differentiate into neurons and glia under standard *in vitro* conditions. Transplanted NPCs integrate into the murine brain and differentiate into neurons, including some TH^+^ neurons.
	Bieri et al. (2019) [164]	*In vitro* model of PFF-induced α-syn aggregation using LRRK2-hiPSC-derived neurons	Strong interaction between LRRK2 and α-syn in PFF model of transmission and aggregation.
	Coremblum et al. (2023) [165]	PD-hiPSC-derived midbrain DA neu-rons	PD neurons show increased α-syn accumulation, altered electrophysiological profiles and increased basal autophagy.
	De Rus Jacquet et al. (2023) [97]	hiPSC-derived 3D model of BBB incorporating astrocytes differentiated from LRRK2-hiPSCs or from isogenic and non-isogenic control hiPSCs	Proinflammatory astrocytes fail to support the formation of a functional barrier.
	Kim et al. (2023) [128]	Modified PD-hiPSC-NPC-derived mid-brain DA neurons that respond to blue light stimulation with α-syn aggregation	Rapid induction of α-syn aggregation and toxicity in DA neurons and midbrain organoids through an assisted α-synuclein aggregation induction system (OASIS).
	Kumar et al. (2023) [166]	GBA1/PD-DA neurons	β-glucocerebroside deficiency leads to steady-state generation of glucosylsphingosine in mutant neurons, which results in aberrant mTORC1 activation and suppresses the ability of DA neurons to clear accumulating α-syn.
	Yao et al. (2023) [167]	LRRK2- and healthy control-hiPSC-DA neurons	The LRRK2 mutation promotes endoplasmic reticulum stress, ROS release and neural cell death.
	Bayati et al. (2024) [168]	hiPSC-derived DA neurons exposed to α-syn PFFs and exposed to IFN-γ	Immune stressors cause lysosomal dysfunction, leading to inclusion formation. Coadministration of PFF and IFN-γ results in the formation and long-term maintenance of PFF^+^ inclusions.
	Bernal-Conde et al. (2024) [169]	3xSNCA-hiPSC, an isogenic SNCA knock-out hiPSC line and a control wild-type hiPSC line transplanted into 6-OHDA-lesioned substantia nigra pars compacta	Supportive role of substantia nigra pars compacta for early TH^+^ neuron differentiation, but SNCA-altered α-syn dosage negatively impacts TH^+^ DA neuron maturation.
	Bhatia et al. (2024) [170]	LRRK2-hiPSC and hiPSC-NPC-derived neurons (*vs.* isogenic controls)	Reduced retrograde trafficking of lysosomes in the axons of mutant neurons. Transient increase in lysosomes at axotomy injury sites in mutant neurons.
	Frattini et al. (2024) [171]	Healthy control and GBA1-hiPSC-derived midbrain organoids containing DA neurons and glia	A reduction in glucocerebroside activity accelerates α-syn pathology by promoting fibrillary α-syn deposition.
	Hembach et al. (2024) [172]	Engrailed-1 KO-hiPSC lines induced to differentiate into neurons	Transcriptomic analysis of hNPCs unveils altered cilia-associated pathways. Reduced mitochondrial respiration in EN-1 KO hNPCs. Shorter neurites, reduced mitochondrial respiration and mitochondrial complex I abundance in fully differentiated neurons.
	Kapucu et al. (2024) [173]	hiPSC-derived cortical networks	Loss of presynaptic proteins and alterations in calcium oscillation and mitochondrial motility during different phases of α-syn aggregation.
	Morrone Parfitt et al. (2024) [174]	DJ1 KO-hiPSC-derived midbrain organoid models	Impaired capacity of the astrocytes to provide metabolic support and acquisition of a proinflammatory phenotype. Impaired lysosomal proteolysis, with AGE accumulation, increased α-syn phosphorylation and protein aggregation.
HD	Camnasio et al. (2012) [175]	HD-hiPSCs and their differentiation to neurons	Increased lysosomal activity in mutant cells.
	Mattis et al. (2012) [176]	14 HD-hiPSC lines with different CAG repeat lengths and control-hiPSC lines	Altered electrophysiology, metabolism, cell adhesion and cell death of cells with medium and longer CAG repeat expansions.
	Ring et al. (2015) [177]	HD-hiPSCs and HD-NSCs (*vs.* corrected isogenic controls)	Transcriptomic analysis of HD cultures *vs.* controls. TGF-β and Netrin-1 as top dysregulated pathways.
	Benraiss et al. (2016) [94]	mHTT-hESC-derived glial progenitor cells and controls. Human glial progenitor cells transduced to express mHTT and their controls	Causal contribution of glia carrying mHTT to HD.
	Lopes et al. (2016) [178]	hESC-derived NSCs	Dominant-negative effect of mHTT on spindle orientation in dividing neural cells and changes in the distribution of dynein, p150*^Glued^* and the large nuclear mitotic apparatus protein NuMA.
	Nekrasov et al. (2016) [179]	Medium spiny neurons derived from HD-hiPSCs, healthy control-hiPSC and a hECS line	mHTT protein aggregation, enhanced Ca^2+^ entry, increased number of lysoso-mes/autophagosomes and enhanced neuronal cell death during cell aging in HD cultures.
	Baronchelli et al. (2017) [180]	Control- and HD-hiPSCs with various CAG repeat lengths differentiated towards medium sized spiny neurons	Gain of methylation during differentiation of HD-hiPSCs. Hypermethylation in genes associated with neuronal development and differentiation; hypermethylation of WD repeat-containing protein 5 in HD-hiPSCs.
	Khoshnan et al. (2017) [181]	hESC-derived DA neurons	Aggregation of the amyloidogenic exon-1 fragment of mHTT activates the expression of neuronal IL-34. Knockdown or blocking the activity of IκB kinase beta (IKKβ) prevents the aggregation of the mHTT fragment and IL-34 production.
	Lim et al. (2017) [182]	Healthy control- and HD-hiPSC-derived neural cultures (neurons, glia and neural progenitors)	Decreased expression in HD cultures of genes associated to neuronal development and maturation, glutamate and GABA signaling, axonal guidance and calcium influx.
	Lim et al. (2017) [183]	Healthy control- and HD-hiPSC-derived BMECs	Aberrant angiogenesis and barrier properties of HD-BMECs. Altered WNT signaling and abnormal BMEC differentiation and maturation.
	Conforti et al. (2018) [184]	Control- and HD-hiPSCs differentiated to striatal and cortical neurons and to 3D organoids	mHTT leads to defects in cortical and striatal progenitor and neuronal specification and neuronal maturation. Neurodevelopmental alterations. Reduced expression of genes related to neuronal migration and differentiation.
	Naphade et al. (2018) [185]	HD-, isogenic- and control corrected-NSCs derived from HD-hiPSC	Altered expression of MMP-2 and MMP-9, MMP3/10 and MMP14 in HD-NSCs. Decreased levels of TIMP-1 and TIMP-2. Association of TIMP-1 with mHTT aggregates in HD-NSCs. Upregulation of TGF-β in HD-NSCs (neuroprotective response).
	Ruzo et al. (2018) [186]	Differentiation to neurons of a collection of HD-hESCs that only differ in the length of the CAG repeat	Chromosomal instability and failed cytokinesis over multiple rounds of DNA replication lead to the emergence of giant multinucleated neurons and neural progenitors, in numbers that are proportional to CAG repeat length.
	García et al. (2019) [187]	HD-hiPSC-derived astrocytes	Altered electrophysiology and reduced cell membrane capacitance. Reduced support for the maturation and survival of hiPSC-derived neurons.
	Goodnight et al. (2019) [188]	Astrocyte differentiation of PSC-NPCs from WT and transgenic Rhesus macaque	Genome-wide alterations in gene expression across differentiation. Altered chromatin accessibility across HD astrocyte differentiation. Premature upregulation of astrocyte differentiation pathways without reaching full maturation and causing early and progressive impairments.
	Kedaigle et al. (2020) [189]	HD- and non-disease-hiPSC-derived neural populations and medium spiny neuron-like cells	Decreased ATP, reduced expression of glycolytic enzymes and increased oxidative phosphorylation in HD-derived cultures of neurons and neural precursors.
	Akimov et al. (2021) [190]	Control- and HD-hiPSC-derived im-immortalized striatal precursor neurons (ISPNs) that can be differentiated to medium spiny neuron-like cells	Decreased viability of HD-ISPNs exposed to glutamate. Altered development, cell-to-cell signaling and metabolic pathways.
	Sorek et al. (2021) [191]	NPCs derived from HD-hiPSCs and iso-genic control-hiPSCs	Single cell-RNAseq demonstrates that mutant HD cells are more heterogeneous than their isogenic controls.
	Linville et al. (2022) [192]	Juvenile and adult onset HD-hiPSC- and isogenic and non-isogenic control-hiPSC-derived BMECs	Decreased efflux activity and altered responses to angiogenic factors, oxidative stress and osmotic stress. Increased immune cell adhesion.
	Reyes-Ortiz et al. (2022) [193]	HD-hiPSC-derived astrocytes	Altered expression of astrogliogenesis transcription factors and altered astrocyte maturation and glutamate signaling.
	Gasser et al. (2023) [194]	Microglia derived from HD-hiPSCs and isogenic control	Increased phagocytic and migratory activity of HD microglia, with significantly higher levels of proinflammatory cytokine release.
	Hernandez et al. (2023) [195]	BMECs and astrocytes derived from HD-hiPSC and non-diseased hiPSC	Dysregulated expression of matrix-interacting genes in HD astrocytes and BMECs. Aberrant interactions at the extracellular matrix/integrin interface weaken BBB fidelity.
ALS	Lenzi et al. (2015) [196]	ALS (mutFUS)-hiPSCs	FUS localizes into stress granules following stress induction.
	Guo et al. (2017) [125]	Control- and ALS (mutFUS)-hiPSC-derived MNs	Cytoplasmic accumulation of mutFUS, hypoexcitability and axonal transport defects. Small reduction in levels of acetylated α-tubulin in mutFUS cultures.
	Hall et al. (2017) [197]	Control- and ALS (mutVCP)-hiPSC-derived MNs and astrocytes	Increased cytoplasmic TDP-43 and endoplasmic reticulum stress in mutant MNs, followed by mitochondrial dysfunction and oxidative stress and leading to ultimate synaptic pathology and cell death. Impaired ability of mutant astrocytes to support MN survival. Increased risk of cell death of the mutant astrocytes compared to controls.
	Qian et al. (2017) [198]	sALS- and healthy control-hiPSC-derived spinal neural progenitors transplanted into mice and differentiated to astrocytes	Degeneration of MNs that are adjacent to the sALS-astrocytes and mouse behavioral deficits. In addition, loss of non-MNs, at even earlier stages.
	Kiskinis et al. (2018) [199]	ALS (mutSOD1)- and isogenic control-hiPSC-derived MNs	Functional optogenetic screening. ALS-MNs fire differently from isogenic controls. Deficit in the Kv7 potassium current in mutant cells.
	Selvaraj et al. (2018) [98]	MNs derived from hiPSCs from patients with mutC9ORF72 and from their corrected isogenic controls	Mutations in C9ORF72 linked to altered AMPAR expression and vulnerability to excitotoxicity.
	Birger et al. (2019) [200]	hiPSC-derived astrocytes from ALS patients carrying C9ORF72 mutations and from non-affected donors	Mutant astrocytes are toxic to MNs through soluble factors and show reduced secretion of several antioxidant proteins. Increased astrocytic oxidative stress and senescence. Conditioned medium from mutant astrocytes induces increased oxidative stress in MNs.
	Melamed et al. (2019) [201]	hiPSC-derived MNs with reduced levels of TDP-43	Inhibition of axonal regeneration of hiPSC-derived MNs. Reduced stathmin-2 expression and enhanced neuronal vulnerability.
	Dafinca et al. (2020) [202]	MNs derived from hiPSCs from ALS patients carrying TDA-43 or C9ORF72 mutations	Increased Ca^2+^ release; impaired mitochondrial Ca^2+^ uptake, contributing to glutamate excitotoxicity.
	Garone et al. (2020) [203]	Control- and ALS (mutFUS)-hiPSC-derived MNs	Altered DNA binding of mutFUS. Upregulation of proteins involved in catabolic processes and oxidation–reduction and downregulation of proteins involved in neuron development and cytoskeleton organization in mutant MNs.
	Smethurst et al. (2020) [204]	hiPSC-derived MNs and astrocytes exposed to serially passaged sALS postmortem tissue extracts	Lower TDP-43 seeded aggregation in astrocytes than in MNs. TDP-43 preferentially spreads from MNs to astrocytes. Neuroprotection conferred by astrocytes against seeded aggregation within MNs by reducing TDP-43-mediated cell toxicity.
	Hartung et al. (2021) [16]	NPCs and MNs derived from an ALS patient with mutated FUS	Changes in DNA methylation associated to mutFUS. Higher DNMT expression and higher methylation of the proximal FUS gene promoter in differentiated mutant MNs.
	Maor-Nof et al. (2021) [205]	MNs derived from ALS-hiPSC carrying C9ORF72 mutations and from isogenic controls	P53 activation and upregulation of key P53-target genes in mutant neurons, inducing DNA damage and apoptosis.
	Namboori et al. (2021) [92]	MNs derived from hiPSCs from ALS patient with mutSOD1 and from isogenic controls	Mutant MN degeneration. Identification of transcription factors associated with MN degeneration in ALS.
	Ren et al. (2021) [130]	Neuronal differentiation of control-, ALS- and HD-hiPSCs to NPCs and to MNs	Establish protocols to generate long-term preservable NPCs from hiPSCs and to induce transcription factor-mediated MN differentiation. MNs differentiated from ALS-hiPSCs display reduced soma size following withdrawal of neurotrophic factors (BDNF, GDNF) and are more vulnerable to this stress state, with extensive cell death.
	Szebényi et al. (2021) [117]	Cerebral organoid slice model obtained from hiPSCs from ALS patients carrying a mutC9ORF72	Early ER stress response in astrocytes and increased poly(GA) levels, DNA damage and cell death of deep layer cortical neurons. Cell subtype-selective susceptibilities. The mutation contributes to UPR activation, stress granule and P62 autophagy marker increases.
	Ziff et al. (2021) [206]	ALS-hiPSC-derived astrocytes	Decreased intron retention in ALS-hiPSC-derived astrocytes, with overexpression of genes related to cell adhesion, the stress response and immune activation. Decreased intron retention is associated with increased cytoplasmic expression of transcripts and proteins regulating reactive transformation of astrocytes. Enhanced nonsense mediated decay in the cytoplasm.
	Almad et al. (2022) [207]	Control-, sALS- and fALS (mutSOD1)-hiPSC-derived astrocytes. Co-culture with control hiPSC-MNs	Increased Cx43 expression; Cx43 hemichannels, enriched at the membrane of ALS-astrocytes, participate in ALS astrocyte-mediated neuronal hyperexcitability and decreased survival. ALS-astrocyte neurotoxicity toward MN and non-MN populations.
	Günther et al. (2022) [208]	Spinal MNs derived from control- and ALS (mutSOD1)-hiPSCs	Loss of mitochondrial integrity precedes elevated levels of aggregated SOD1 in mutant MNs.
	Cheesbrough et al. (2023) [209]	hiPSC-neuro-muscular disease model: hiPSC-myofibers innervated by mutant TDP-43- and corrected control hiPSC-MNs in 96-well assay plates. MNs respond to optogenetic stimulation	Model as a readout of neuromuscular transmission. Weaker myofiber contractions, reduced axonal outgrowth and reduced number of neuromuscular synapses with mutant neurons.
	Massih et al. (2023) [210]	hiPSC-derived MNs from ALS patients with mutSOD1 co-cultured with 3D skeletal muscle tissue	Reduced neuromuscular coupling and muscle contraction in co-cultures with mutant neurons.
	Stoklund Dittlau et al. (2023) [211]	Astrocytes derived from hiPSCs carrying FUS mutations and from their isogenic controls in a human motor unit microfluidics model (MNs and myotubes)	Mutant astrocytes show increased reactivity and secretion of inflammatory cytokines. Cytotoxic effect of mutant astrocytes on MN-neurite outgrowth, neuromuscular formation and functionality.
	Tsioras et al. (2023) [212]	hiPSC-derived MNs from patients with mutated SOD1 and from non-disease controls	Altered interactome of VCP in mutSOD1 MNs, contributing to impaired protein degradation. Enhanced accumulation of disordered mutSOD1 in mutSOD1-MNs as they age.
	Baskerville et al. (2024) [213]	sALS-derived neurons	Nuclear pore complex injury cascades associated with a nuclear influx of CHMP7 due to defects in nuclear–cytoplasmic cellular compartmentalization and specific nuclear membrane proteins.
	Dafinca et al. (2024) [214]	hiPSC-derived MNs from ALS patients with mutated TDP-43 (*vs.* corrected isogenic controls)	Reduced ATP production and expression of the motor proteins DCTN1 and dynein, with significant downregulation of retrograde axonal transport.
	Guo et al. (2024) [215]	Control- and C9ORF72 knock-down- hiPSC-derived astrocytes, MNs and spinal cord organoids	Higher levels of inflammatory cytokines in mutant astrocytes and MNs. Additionally, cells other than astrocytes play an important role in C9ORF72-ALS-induced neuroinflammation
	Hruska-Plochan et al. (2024) [216]	Self-renewing hiPSC-derived, colony morphology NSCs (iCoMoNSCs) differentiated into electrophysiologically connected neurons and glia	Misregulation of RNA targets. Marked NPTX2 accumulation, linked to neurotoxicity.
	Keeley et al. (2024) [217]	sALS-hiPSC-derived neurons	Overactivation of the ESCRT-III nuclear surveillance pathway, dependent on the ESCRT-III protein CHMP2B.

**Table 2:** Stem cell-based preclinical and clinical studies on NDs.

Disease	Reference	Stem Cell	Animal Model/Trial	Administration	Main Outcomes
AD (preclinical studies)	Park et al. (2020) [233]	MSCs	AD mouse model	Intravenous	Reduced amyloid plaques, improved PET imaging findings.
	Cone et al. (2021) [234]	MSC-derived extracellular vesicles	5XFAD mouse model	Intravenous	Modulated neuroinflammation and synaptic function. Ameliorated AD-like phenotypes.
	Jeong et al. (2021) [235]	Gene-modified MSC-derived extracellular vesicles	Aβ1-42-injected AD mouse model	Intravenous	Expression of neprilysin showed enhanced clearance of amyloid-beta.
	Neves et al. (2021) [236]	MSCs	3xTg-AD mice	Intravenous injection	Reduced Tau phosphorylation, anti-inflammatory effects and modulation of AD pathology.
	Ali et al. (2023) [237]	Wharton’s Jelly MSCs	AD rat model	Intravenous	Highlighted the role of RYR3 gene in MSC-mediated therapy.
	Ban et al. (2023) [238]	hNSCs and Microglia	AD mouse model	Intracerebral	Synergistic approach showed enhanced outcomes in AD pathology.
	Gao et al. (2023) [239]	NSCs-derived extracellular vesicles	5XFAD mouse model	Intravenous	Mitigated AD-like phenotypes with reduced amyloid and improved cognition.
	Mishra et al. (2023) [240]	HSCs and Progenitor Cells	5XFAD mouse model	Intracerebral	Reduced neuroinflammation and amyloid pathology with behavioral improvement.
	Yoo et al. (2023) [241]	Circulation-derived myeloid cells (CDMCs)	5XFAD mouse model	Hemato-poietic cell transplantation	Restored microglial Trem2 function, improved AD pathology.
	Ebrahim et al. (2024) [242]	MSC-derived exosomes	AD rat model	Intravenous	Emphasized role of the PI3K/Akt/mTOR path-way in reducing AD pathology, modulating autophagy, and decreasing inflammation.
	Ji et al. (2024) [243]	iNSCs	APP/PS1 mouse model	Intracerebral	Reduced amyloid plaques, improved behavior.
	Lin et al. (2024) [244]	MSC-derived extracellular vesicles	sAD mouse model	Intravenous	Reduced neuroinflammation via NLRP3/GSDMD pathways. Focus on inflammatory cascades in sporadic AD.
	Milazzo et al. (2024) [245]	HSC gene therapy	5XFAD mouse model	Intracerebral	Significant reduction in Tau pathology.
AD (clinical studies)	Duma et al. (2019) [246]	Adipose-derived stromal vascular fraction (ADSVF)	Phase I Clinical Trial	Intracerebroventricular injection	Well-tolerated; cognitive stability or improvement in AD patients.
	Kim et al. (2021) [247]	Human umbilical cord MSCs	Phase I Clinical Trial	Intracerebro-ventricular injection	Safe administration, potential efficacy noted in AD patients.
	Brody et al. (2023) [248]	Lomecel-B MSCs	Phase I Clinical Trial	Intravenous	Safe administration with potential cognitive benefits.
	Xie et al. (2023) [249]	Adipose MSC-derived Exosomes	Phase I/II Clinical Trial	Intravenous	Focus on neuroinflammation and amyloid pathology, cognitive assessment and biomarker analysis in mild-moderate AD.
	NCT04388982 [250]				
	NCT02833792 (2016–Ongoing) [251]	hMSCs	Phase II Clinical Trial	Intravenous	Preliminary efficacy of hMSCs versus placebo in subjects with AD-related dementia, as evidenced by neurologic, functional and psychiatric endpoints.
	NCT04482413 (2022–Ongoing) [252]	Autologous adipose tissue-derived MSCs	Phase I/II Clinical Trial	Intravenous delivery	Safety and efficacy evaluation in AD patients, focus on amyloid reduction and cognitive function improvement.
	NCT05667649 (2023–Ongoing) [253]	Ex vivo-expanded adipose-derived stem cells (RB-ADSCs)	Phase I Clinical Trial	Intracerebro-ventricular injection	Recruiting; primary objectives are evaluating adverse events and dose-limiting toxicities along with Tau and Aβ levels.
PD (preclinical studies)	Chen et al. (2020) [254]	MSC-derived exosomes	Mouse model	Intracerebral injection to the striatum	Induced autophagy and repaired Parkinsonian model.
	Doi et al. (2020) [255]	iPSCs	PD rat model	Striatal transplantation	Behavioral improvement leading to the beginning of clinical trials.
	Xue et al. (2021) [256]	MSC-derived exosomes	Mouse model	Inperitoneal	Enhanced angiogenesis of BMECs and therapeutic potential.
	Li et al. (2022) [257]	Genetically engineered MSCs	PD rat and Rhesus monkey models	Intracerebral	Demonstrated dopamine synthesis and motor recovery.
	Xu et al. (2022) [258]	hiPSC-based replacement therapy	Mouse model	Intracerebral	Promotion of midbrain DA neuronal differentiation and corrected motor PD deficits.
	Hamedi et al. (2023) [259]	Adipose tissue-derived MSC transplantation	Rat model	Intracerebral	Promoted DA neurons and improved memory.
	López-Ornelas et al. (2023) [260]	hESC-derived DA neurons, midbrain DA neuron transplantation	Parkinsonian monkeys	Intracerebral	Improvement in motor function, potential for functional recovery viability and integration of transplanted neurons.
	Lyu et al. (2023) [261]	miR-210-5p targeting SMAD4 and SUFU for differentiation of hiPSCs	PD rat model	Intracerebral	Promoted DA neuron differentiation.
	Zheng et al. (2023) [262]	hiPSC-derived midbrain organoids	6-OHDA PD mouse model	Intracerebral	Restoration of motor function. Organoids exhibit functional connectivity with host brain.
	Daadi et al. (2024) [263]	iPSC-derived DA neuron transplantation combined with physical and cognitive training	Parkinsonian marmosets	Intracerebral	Better graft integration and improved functional outcomes. Synergy between neuronal transplantation and rehabilitation.
	Ge et al. (2024) [264]	HSC-based microglia delivery of GDNF	MPTP PD mouse model	Bone marrow transplantation	Systemic approach leading to long-term benefits to CNS.
	Narasimhan et al. (2024) [265]	Cryogel microcarriers loaded with GDNF	Rat model	Intra-striatal transplantation	Enhanced engraftment of DA neurons.
PD (clinical studies)	Schweitzer et al. (2020) [266]	Personalized iPSC-derived dopamine progenitors	PD patient	Intracerebral	Functional recovery and motor improvement.
	Jamali et al. (2021) [267]	MSCs differentiated into NSCs	Phase I Clinical trial	Injection of Umbilical cord-derived MSCs	Protocol development for safety evaluation.
	NCT03684122 [268]				
	Piao et al. (2021) [269]	hESC-derived midbrain dopamine progenitors (MSK-DA01)	Phase I/II Clinical trial	Direct DA progenitor transplantation	Demonstrated safety and early signs of efficacy.
	NCT04802733 [270]				
	NCT05897957 [271]				
	Shigematsu et al. (2022) [272]	Autologous adipose tissue-derived stem cells	PD patients	Intravenous	Repeated administration was safe and feasible.
	Kirkeby et al. (2023) [273]	hESC-derived product (STEM-PD)	Preclinical assessment and Phase I Clinical trial	Intraputamenal trans-plantation	Preclinical validation and assessment of the safety and feasibility of intraputamenal STEM-PD transplantation.
	NCT05635409 [274]				
	Jiang et al. (2024) [275]	hNSCs	Phase I Clinical trial	Intranasal transplantation	Demonstrated safety and preliminary efficacy.
	Park et al. (2024) [276]	Dose-ranging assessment of hESC-derived DA progenitors	Preclinical assessment	Intracerebral	Established preclinical safety for clinical trials.
	Zhuo et al. (2024) [277]	Hypoxia-preconditioned olfactory mucosa MSCs	Mouse models/Phase I Clinical trial	Intracerebral	Improved neural functional recovery mediated by TGF-β1.
HD (preclinical studies)	Cho et al. (2019) [278]	Genetically modified NPCs derived from Rhesus monkey iPSCs	HD mice	Intracerebral	Ameliorated Huntin-gton’s symptoms, increased lifespan, and promo-ted differentiation into GABAergic neurons.
	Yoon et al. (2020) [279]	Clinical-grade human NSC line (CTX0E03)	Quinolinic acid-lesioned rodents	Intracerebral	Behavioral and pathological deficits rescued.
	Islam et al. (2021) [280]	hESCs	AAV2-Htt171-82Q transfected rats	Intracerebral	Motor dysfunction alleviated.
	Wenceslau et al. (2022) [281]	Immature dental pulp stem cells	3-NP rat model	Intravenous	Restored neurotrophic factors such as BDNF, promoted neuroprotection and neurogenesis in the striatum and cortex.
HD (clinical studies)	Macedo et al. (2021) [282] 2/25/2025 10:58:00	Immature hu-man dental pulp stem cells (Nes-taCell HDTM)	Phase II Clinical trial Phase III Clinical trial	Intravenous	Initial safety and feasibility study in Huntington’s disease patients. Significant improvement in motor scores and functional capacity compared to placebo.
	NCT03252535 [283]				
	NCT04219241 [284]				
	NCT06097780 [285]				
ALS (preclini-cal studies)	Forostyak et al. (2020) [286]	iPSC-derived neural precursors	SOD1G93A-transgenic rats	Intraspinal	Preserved neural plasticity and perineuronal nets. Transplanted cells pro-moted regeneration and network stability.
	Yamashita et al. (2020) [287]	Multilineage–differentiating stress–enduring (Muse) cells	G93A-transgenic ALS mice	Intravenous	Improved motor function and reduced inflammation.
	Giunti et al. (2021) [288]	MSC-derived small extracellular vesicles	SOD1G93A mice	Intravenous	Modulation of neuro-inflammation via miRNAs.
	Laperle et al. (2023) [289]	iPSC-derived NPCs	SOD1G93A ALS rat model	Intracranial	Protection in ALS and retinal degeneration via GDNF secretion.
ALS (clinical studies)	Berry et al. (2019) [290]	NurOwn (autologous MSC-derived cells)	Phase II Clinical trial	Combined intrathecal and intra-muscular administration	Demonstrated safety; mixed efficacy results in biomarker and functional outcome monitoring.
	NCT03280056 [291]				
	Petrou et al. (2021) [292]	Autologous MSCs	Phase II Clinical trial	Intrathecal injections	Safe and feasible improvement in biomarkers and ALS functional rating scale.
	Nakamura et al. (2023) [293]	iPSC-derived MNs	Genetic ALS research		Identified genetic factors in ALS; correlated with iPSC MN behavior.
	Nam et al. (2023) [294]	Autologous BM-derived MSCs (Neuronata-R^®^)	Clinical (surveillance)	Intrathecal	Long-term survival benefit observed.
	NCT04651855 (2020–Ongoing) [295]	Wharton’s Jelly MSCs	Phase I/II Clinical trial	Intrathecal	Evaluation on the immune system of patients with ALS.
	Imamura et al. (2024) [296]	iPSC drug repurposing study (bosutinib)	Phase II Clinical trial	Oral administration	Evaluation of bosutinib’s efficacy using iPSC models.
	NCT04849065 (2021–Ongoing) [297]	Autologous bone marrow mono-nuclear cells	Phase II Clinical trial	Intramuscular injection	Expected positive effect on the natural loss of motor units in ALS patients.
	NCT02478450 (2024–Ongoing) [298]	Human glial restricted progenitor cells (hGRPs; Q-Cells^®^)	Phase I Clinical trial	Intraspinal transplantation	Safety and preliminary efficacy in ALS patients.

## Data Availability

No new data were created or analyzed in this study. Data sharing is not applicable to this article.

## Note

This article is dedicated to the memory of Francisco Durán Fernández and to that of Mercedes Schembri-Wismayer.

## Abbreviations

The following abbreviations are used in this manuscript:

ND	Neurodegenerative disease
(s/f) AD	(sporadic/familial) Alzheimer’s Disease
PD	Parkinson’s Disease
HD	Huntington’s Disease
ALS	Amyotrophic Lateral Sclerosis
Aβ	Amyloid beta
APP	β-Amyloid precursor protein
NFTs	Neurofibrillary tangles
α-syn	α-Synuclein
p-Tau	Phosphorylated Tau
MN	Motor neuron
HTT	Huntingtin
BBB	Blood–brain barrier
SVZ	Subventricular zone
SGZ	Subgranular zone
DG	Dentate gyrus
NSCs	Neural stem cells
DCX	Doublecortin
OB	Olfactory bulb
DGC	Dentate granule cell
PS1	Presenilin-1
DA	Dopaminergic
NPCs	Neural progenitor cells
(h)iPSC	(human)induced pluripotent stem cells
ESCs	Embryonic stem cells
BMECs	Brain microvascular endothelial cells
MSCs	Mesenchymal stem cells
HSCs	Hematopoietic stem cells
EVs	Extracellular vesicles
ASOs	Antisense oligonucleotides
